# Harmful dimensions of medical culture across physician subgroups: a cross-sectional study of 1002 physicians predominantly practising in Belgium

**DOI:** 10.1136/bmjopen-2026-124359

**Published:** 2026-10-20

**Authors:** Emilie Banse, Moïra Mikolajczak, Marie Bayot, Anne-Laure Lenoir, Philippe De Timary

**Affiliations:** 1 Psychological Sciences Research Institute, Department of Psychology, Catholic University of Louvain, Louvain-la-Neuve, Belgium; 2 Department of Adult Psychiatry, Cliniques universitaires Saint-Luc, Brussels, Belgium; 3 Department of Psychology, Faculty of Medicine, University of Namur, Namur, Belgium; 4 Department of General Practice/Family Medicine, Liège University Hospital Sart Tilman Site, Liège, Walloon Region, Belgium

**Keywords:** Burnout, Professional, Education, Medical, Physicians

## Abstract

**Abstract:**

**Objectives:**

To examine whether physicians’ endorsement of harmful dimensions of medical culture (HDMC)—namely Physicians’ Professional Commitment, the Myth of the Invulnerable Physician and Stigma towards Burnout—varies according to demographic and professional characteristics.

**Design:**

Cross-sectional online survey study.

**Setting:**

Primary and secondary care settings, predominantly in French-speaking Belgium, with additional recruitment in France.

**Participants:**

1002 French-speaking physicians in clinical practice, including general practitioners, specialists, residents and attending physicians.

**Primary and secondary outcome measures:**

The outcomes examined were scores on the three dimensions of the revised 20-item HDMC model: Physicians’ Professional Commitment, the Myth of the Invulnerable Physician and Stigma towards Burnout. Analyses were exploratory. The specific subgroup comparisons and multivariable analyses reported in this study were not preregistered.

**Results:**

In multivariable analyses of 941 complete cases, higher Physicians’ Professional Commitment scores were associated with female gender (β=0.11), being a specialist rather than a general practitioner (β=0.15) and longer weekly working hours (β=0.29). Higher Myth of the Invulnerable Physician scores were associated with younger age (β=−0.32), female gender (β=0.20) and longer weekly working hours (β=0.08). Higher Stigma towards Burnout scores were associated with younger age (β=−0.13), being a specialist rather than a general practitioner (β=0.08) and longer weekly working hours (β=0.11). The models explained modest proportions of variance (adjusted R²=0.05–0.15).

**Conclusions:**

Physicians’ endorsement of harmful dimensions of medical culture varied across demographic and professional characteristics, with distinct patterns across the three HDMC dimensions. Although associations were generally modest and subgroup differences should be interpreted cautiously, the findings highlight heterogeneity in how specific medical cultural norms are endorsed. Further research in independent and diverse samples is needed to examine how individual, professional, organisational and cultural contexts may shape these patterns.

**Study preregistration:**

The broader research project was preregistered on the Open Science Framework (OSF) https://doi.org/10.17605/OSF.IO/NTSZC

**STRENGTHS AND LIMITATIONS OF THIS STUDY**

- The study included a large and professionally diverse sample of over 1000 physicians across different specialties and career stages.
- The study used a multidimensional measure of medical culture based on the previously developed harmful dimensions of medical culture scale, with a revised 20-item measurement model used in the present analyses.
- The cross-sectional design precludes causal or temporal inferences.
- Convenience sampling may have introduced selection bias and limits the generalisability of the findings.
- Full scalar measurement invariance was not supported across the principal physician subgroup comparisons examined, although configural, metric and partial scalar invariance were achieved.

## Introduction

 Physician well-being is a growing public health concern, with consistently high rates of burnout, depression, suicidal ideation and maladaptive health behaviours reported across the profession.^1–5^ These issues not only impact individual physicians but also jeopardise workforce sustainability and the quality of patient care.^6 7^ While structural and occupational stressors have long been recognised as key contributors to distress in the profession, increasing attention has turned to medical culture, that is, the implicit norms, values and expectations that shape professional identity and behaviour, as a critical yet underexamined contributor to physician distress and poor self-care.^8–11^

Recent research has conceptualised three interrelated harmful dimensions of medical culture (HDMC) with particular relevance to physician well-being.^12^ (1) Physicians’ Professional Commitment captures the extent to which physicians internalise their professional role as central to their personal identity and daily life. It reflects the perception that a successful medical career necessitates significant personal sacrifice and is often accompanied by a persistent sense of work-life imbalance. Importantly, professional commitment is not inherently harmful and can be a source of meaning, fulfilment and high-quality patient care. In the HDMC framework, the potentially harmful aspect refers specifically to forms of commitment in which professional identity and obligations become highly dominant and are accompanied by persistent self-sacrifice, work prioritisation and interference with other life domains. (2) The Myth of the Invulnerable Physician reflects the enduring belief that physicians should be impervious to personal struggle. It encompasses the idea that the roles of physician and patient are inherently incompatible, and that physicians bear a moral obligation to prioritise the needs of patients and colleagues above their own. (3) Stigma towards Burnout reflects negative attitudes and perceived social judgement surrounding the experience or disclosure of burnout within the medical profession. These medical cultural norms are implicitly conveyed through medical training^13^ and further reinforced by organisational environments that perpetuate unrealistic expectations.^11^ They tend to co-occur and amplify one another.^12^ Critically, physicians’ endorsement of the HDMC has been linked to adverse outcomes including burnout, depression, avoidance of care, presenteeism and self-medication.^12 14^ Examining whether these cultural norms vary according to physicians’ demographic and professional characteristics may therefore provide a more differentiated understanding of how medical culture is endorsed across the profession. Yet, whether endorsement of these harmful cultural norms varies across physician subgroups remains largely unexplored. Although related literature suggests that factors such as gender, specialty, career stage and personal life context may be associated with how physicians relate to professional norms, these differences have not been directly examined using the HDMC framework. Examining such variation may also help contextualise previously documented differences in physician well-being across demographic and professional groups.^15^

In response to this, this study explores whether the endorsement of three HDMCs (Physicians’ Professional Commitment, the Myth of the Invulnerable Physician and Stigma towards Burnout) varies according to various demographic and professional characteristics. Specifically, we examine variations in relation to gender, age, parental status, relationship status, physician type (general practitioner (GP) vs specialist), career status (resident vs attending physician) and weekly working hours. Given the absence of prior empirical work on this specific topic, the present study is post-hoc exploratory and hypothesis-generating rather than confirmatory. We examine whether HDMC endorsement varies across physician subgroups without prespecified hypotheses regarding the direction or strength of these associations.

## Methods

This study is part of a broader research project investigating the interplay between psychological distress, health behaviours and medical culture.

Two previous studies have used data from the same broader research project. The first focused on the development and psychometric evaluation of the HDMC scale and examined its association with physician burnout.^12^ The second examined associations between HDMC scores and a broader range of mental health outcomes and health-related behaviours, including depression, presenteeism, help-seeking, self-diagnosis, self-treatment, psychoactive medication use and alcohol consumption.^14^ In contrast, the present study examines whether HDMC endorsement varies according to physicians’ demographic and professional characteristics, using subgroup comparisons and multivariable regression analyses. Thus, although the three studies share the same underlying sample and originate from the same HDMC item data, their primary research questions, variables of interest and principal analyses are distinct, and the analyses reported in the present study do not duplicate those reported in the previous publications.

The broader research project was preregistered on the Open Science Framework (OSF) before data collection (https://doi.org/10.17605/OSF.IO/NTSZC). The preregistration specified the development and examination of the structure of medical-culture dimensions and their associations with physicians’ mental health and health behaviours, and anticipated exploratory analyses examining whether these associations varied according to sociodemographic and professional characteristics. However, the specific research question addressed in the present study (whether HDMC endorsement differs across physician subgroups) was not preregistered. The subgroup comparisons, variable categorisations and multivariable regression models reported here were also not prespecified in the OSF registration. The analyses reported in the present study should therefore be considered post-hoc exploratory and hypothesis-generating.

### Survey instrument

An online survey was administered to participants, with anonymisation to ensure confidentiality. Data collection and management were facilitated through Research Electronic Data Capture (REDCap) electronic data capture tools.^16^ Participants provided informed consent electronically prior to survey completion. The survey collected personal and professional characteristics, including age, gender, relationship status, parental status, career status, specialty, average weekly working hours and country of residence. Gender was self-reported using the response options ‘woman’, ‘man’ and ‘other’; biological sex was not assessed. The survey also included the HDMC scale,^12^ which assesses three interdependent dimensions of medical culture: Physicians’ Professional Commitment, the Myth of the Invulnerable Physician and Stigma towards Burnout. In the original 21-item HDMC measurement model, Physicians’ Professional Commitment comprised three first-order subfactors (work priority strain, the self-sacrificing nature of medical practice and the physician’s central identity role), the Myth of the Invulnerable Physician comprised two (physician’s moral obligation to patients and colleagues and physician’s discomfort with the patient role) and Stigma towards Burnout comprised two (colleagues’ stigma towards burnout and personal stigma towards burnout).^12^ However, during the additional measurement-invariance analyses conducted as part of peer review, the measurement model was refined post hoc by removing one item from Stigma towards Burnout. In the resulting 20-item model used in the revised analyses, the first-order structure of Physicians’ Professional Commitment and the Myth of the Invulnerable Physician remained unchanged, whereas Stigma towards Burnout was represented directly by its three remaining items. The revised measurement model is presented in online supplemental figure 3.

The items of each dimension are rated on a 5-point Likert scale ranging from ‘strongly disagree’ (1) to ‘strongly agree’ (5). Illustrative items for each dimension include, respectively, ‘My professional life often invades my private life’, ‘Having the role of doctor could become a barrier if I should ever have to adopt the role of patient’ and ‘Physicians who experience burnout are often judged as being weak by their colleagues’.

Composite scores for each dimension were calculated by averaging the corresponding items, with higher scores indicating greater endorsement of the respective cultural norm. The three dimension scores were used as the outcomes in the inferential analyses. For descriptive purposes, a global HDMC score was also calculated as the mean of all 20 items retained in the revised measurement model. However, as the measurement model did not specify an overarching latent global HDMC factor, the global HDMC score was treated as an item-based descriptive composite rather than as an additional inferential outcome.

Given that Physicians’ Professional Commitment, the Myth of the Invulnerable Physician and Stigma towards Burnout comprise 11, 6 and 3 items, respectively, the global score can equivalently be expressed as: (11×Physicians’ Professional Commitment + 6×Myth of the Invulnerable Physician + 3×Stigma towards Burnout)/ 20. Thus, each retained item contributes equally to the global score. No items are reverse scored.

All 1002 participants included in the present study had complete responses to the 20 retained HDMC items; therefore, no imputation or other procedure for handling missing HDMC item responses was required.

The original 21-item structure and its three dimensions were established and psychometrically examined using exploratory and confirmatory factor analyses and internal consistency analyses.^12^ The revised 20-item measurement model and corresponding descriptive global composite used here resulted from the post-hoc model refinement undertaken in the context of the measurement-invariance analyses described below and were therefore not the scoring specification examined in the original psychometric study.

### Study population

The participants were French-speaking physicians in current clinical practice, encompassing GPs, specialist physicians (all specialties) and medical residents (postgraduates in training to become GPs or specialists). Data were collected between 5 September and 5 November 2023. To recruit a broad and professionally diverse convenience sample, we used several approaches to encourage participation. First, the survey was disseminated on social media networks and via word of mouth. Leading medical organisations in Belgium, general medicine organisations and physicians’ magazines relayed the survey, with some additional recruitment in France. Deans of medical faculties at French-speaking universities in Belgium shared the survey via their mailing lists for residents and academic physicians at their respective university hospitals. Medical directorates and councils of hospitals shared the survey among their physicians. Participation was voluntary and without financial incentive.

The present study reports secondary exploratory analyses of an existing dataset collected as part of the broader research project. No separate a priori sample-size calculation was conducted for the specific subgroup comparisons and multivariable analyses reported here. The available sample comprised 1002 physicians with complete data on the 20 HDMC items; analyses were conducted using available data for each subgroup comparison, while the multivariable regression analyses included 940 participants with complete data on all model covariates.

### Statistical analyses

All statistical analyses aimed to identify potential patterns in the endorsement of the HDMC across physician subgroups. Following initial data cleaning, descriptive statistics were computed for all variables, and the internal consistency of the descriptive global HDMC score, the three dimensions and the five first-order subfactors retained in the revised 20-item model was assessed using Cronbach’s alpha. The three HDMC dimension scores constituted the outcomes of the inferential analyses, while the global HDMC score was retained for descriptive purposes only.

Age and weekly working hours were recoded into categories for the purpose of subgroup comparisons, following categories used in related literature^17 18^: age was categorised as <35, 35–44, 45–54, 55–64 and ≥65 years; weekly working hours were categorised as <30, 30–39, 40–50 and >50 hours per week. Table 1 presents physician type using the original multicategory survey response. For the binary physician-type and career-status subgroup comparisons, one participant who selected the combined ‘occupational medicine/insurance physician/school physician/medical consultant’ category was coded as a specialist. Because this participant’s career status was not directly reported, they were assigned to the attending group based on their age category (55–64 years). Thus, the binary comparisons included 646 specialists and 736 attending physicians, whereas the original categories in table 1 included 645 specialists and 735 attending physicians.

**Table 1:** Sociodemographic and work-related characteristics of the sample

Sociodemographic and work-related characteristics*	No. (%)
Age category	
<35 years old	398 (39.7)
Between 35 and 44 years old	216 (21.6)
Between 45 and 54 years old	183 (18.3)
Between 55 and 64 years old	130 (13.0)
≥65 years old	45 (4.5)
Missing	30 (3.0)
Gender	
Woman	647 (64.6)
Man	347 (34.6)
Missing	6 (0.6)
Other	2 (0.2)
Relationship status	
In a relationship	825 (82.3)
Single	175 (17.5)
Missing	2 (0.2)
Parental status	
No	415 (41.4)
Yes	582 (58.1)
Missing	5 (0.5)
Physician (career) status	
Attending specialist	457 (45.6)
Attending general practitioner	278 (27.7)
Resident specialist	188 (18.8)
Resident general practitioner	61 (6.1)
Occupational medicine/insurance physician/school physician/medical consultants	15 (1.5)
Missing	3 (0.3)
Work setting of the (resident) specialist (among specialists, N=645)	
Hospital setting only	560 (86.8)
Both hospital setting and private consultation	60 (9.3)
Private consultation only	13 (2.0)
Missing	12 (1.9)
Specialty of the (resident) specialist (among specialists, n=645)	
Internal medicine	170 (26.4)
Surgery	66 (10.2)
Acute medicine	70 (10.9)
Clinical pathology	18 (2.8)
Anaesthesiology	60 (9.3)
Dermatology	13 (2.0)
Obstetrics and gynaecology	25 (3.9)
Nuclear medicine	8 (1.2)
Physical medicine	15 (2.3)
Paediatrics and neonatology	54 (8.4)
Ophthalmology	10 (1.6)
Otolaryngology	18 (2.8)
Psychiatry	36 (5.6)
Radiology	12 (1.9)
Palliative care	2 (0.3)
Neurology	40 (6.2)
Radiotherapy—oncology	5 (0.8)
Other^†^	5 (0.8)
Missing	18 (2.8)
Type of practice^‡^ of the (resident) generalist (among generalists, N=339)	
General medicine (consultations and home visits)	327 (96.5)
Other institution (eg, family planning centre, nursing home)	52 (15.3)
Hospital setting	14 (4.1)
Non-conventional medicine	11 (3.2)
Work setting^‡^ of the (resident) generalist (among generalists, N=339)	
Multidisciplinary group	150 (44.2)
Monodisciplinary group	107 (31.6)
Works alone	90 (26.5)
Weekly working hours category	
<30 hours	34 (3.4)
Between 30 and 39 hours	121 (12.1)
Between 40 and 50 hours	452 (45.1)
>50 hours	394 (39.3)
Missing	1 (0.1)
Country of residence	
Belgium	922 (92.0)
France	65 (6.5)
Switzerland	2 (0.2)
Other	6 (0.6)
Missing	7 (0.7)

Percentages in the specialist specialty section are based on the 645 physicians classified as attending or resident specialists in table 1; 627 reported a specialty and 18 had missing specialty data.

N=1002.

* Data were missing for some characteristics.

† Includes hospital hygiene, medical information, orthodontics, legal medicine.

‡ Multiple responses were possible.

No, number.

To explore group differences in the three HDMC dimension scores across physician characteristics (age category, gender, relationship status, parental status, career status, physician type and weekly working hours category), we used independent-samples t-tests or one-way analysis of variance (ANOVAs) as appropriate. Cohen’s *d* and eta-squared (η²) were reported as effect-size estimates for two-group and multigroup comparisons, respectively. Prior to conducting these inferential tests, we verified the relevant assumptions. The distribution of HDMC variables was assessed through visual inspection of histograms and Q-Q plots. Homogeneity of variance was examined using Levene’s test. When Levene’s test indicated a violation of the homogeneity of variance assumption, we reported the adjusted test statistics and corresponding p values (Welch’s ANOVA), as appropriate. Independence of observations was assumed by design. To assess the robustness of findings, non-parametric equivalents (Mann-Whitney U and Kruskal-Wallis tests) were also conducted. As results were consistent with those of parametric tests, only the latter are reported for clarity.

Post-hoc comparisons were performed using Bonferroni correction following standard ANOVA where relevant (see online supplemental table 2). When Welch’s ANOVA was used because of unequal variances, Games-Howell post-hoc comparisons were performed. The subgroup comparisons reported in table 2 constituted the principal analyses of physician characteristics. No global multiplicity correction was applied across all subgroup comparisons. Given the exploratory nature of these analyses, interpretation therefore considered effect sizes and the overall pattern of findings rather than individual p values alone.

**Table 2:** HDMC dimension scores according to physicians’ characteristics

	N	Physician’s Professional Commitment (mean, SD)	Test statistic (p value); effect size	Myth of the Invulnerable Physician (mean, SD)	Test statistic (p value); effect size	Stigma towards Burnout (mean, SD)	Test statistic (p value); effect size
Age category			*F*=1.03, p=0.391; η² = 0.004		Welch’s *F*=27.57, p<0.001; η² = 0.111		Welch’s *F*=10.55, p<0.001; η² = 0.040
<35 years old	398	3.67±0.65		3.85±0.64		2.70±0.83	
Between 35 and 44 years old	216	3.66±0.65		3.70±0.74		2.48±0.89	
Between 45 and 54 years old	183	3.69±0.61		3.59±0.72		2.36±0.79	
Between 55 and 64 years old	130	3.75±0.60		3.24±0.84		2.28±0.76	
≥65 years old	45	3.53±0.66		2.97±0.74		2.30±0.82	
Gender							
Man	347	3.64±0.66	t = −1.34, p=0.180; d = −0.09	3.38±0.81	t = −7.95, p<0.001; d = −0.56	2.45±0.83	t = −1.72, p=0.086; d = −0.11
Woman	647	3.70±0.62		3.79±0.68		2.54±0.86	
Relationship status							
Single	175	3.66±0.67	t = −0.24, p=0.808; d = −0.02	3.61±0.80	t = −0.68, p=0.498; d = −0.06	2.52±0.83	t=0.15, p=0.885; d=0.01
In a relationship	825	3.68±0.63		3.65±0.75		2.51±0.85	
Parental status (children to care for)							
Yes	582	3.66±0.62	t = −0.98, p=0.328; d = −0.06	3.52±0.79	t = −6.49, p<0.001; d= −0.41	2.41±0.83	t = −4.34, p<0.001; d= −0.28
No	415	3.70±0.66		3.82±0.66		2.65±0.85	
Physician type							
General practitioner	339	3.48±0.68	t = −6.97, p<0.001; d = −0.49	3.60±0.78	t = −1.49, p=0.135; d= −0.10	2.38±0.85	t = −3.75, p<0.001; d= −0.25
Specialist	646	3.78±0.59		3.67±0.74		2.59±0.84	
Career status							
Attending	735	3.67±0.64	t = −0.75, p=0.456; d = −0.06	3.57±0.78	t = −6.46, p<0.001; d= −0.42	2.50±0.77	t = −4.24, p<0.001; d = −0.31
Resident	249	3.71±0.64		3.88±0.61		2.74±0.77	
Weekly working hours category			Welch’s *F*=43.15, p<0.001; η² = 0.104		*F*=3.07, p=0.027; η² = 0.009		*F*=6.24, p<0.001; η² = 0.018
<30 hours	34	3.33±0.67		3.46±0.76		2.34±0.77	
Between 30 and 39 hours	121	3.35±0.56		3.49±0.73		2.37±0.83	
Between 40 and 50 hours	452	3.59±0.66		3.66±0.76		2.44±0.85	
>50 hours	394	3.91±0.54		3.69±0.74		2.65±0.85	

Depending on the number of groups compared, independent-samples t-tests or one-way ANOVAs were conducted. Welch’s ANOVA was used when the homogeneity of variance assumption was violated. Reported values indicate the test statistic (t or *F*) followed by the corresponding two-sided p value and effect size. Effect sizes are reported as Cohen’s *d* for two-group comparisons and eta-squared (η²) for comparisons involving more than two groups. For two-group comparisons, Cohen’s d was calculated as the first group listed minus the second group listed. One participant selected ‘specialist physician’ in the survey and specified occupational medicine as their specialty. This participant was classified in the occupational medicine category in table 1 but retained as a specialist in the binary physician-type comparison in this table. Their career status was not directly reported and was treated as missing in the attending-versus-resident comparison. The resulting groups comprise 646 specialists and 735 attending physicians.

ANOVA, analysis of variance; HDMC, harmful dimensions of medical culture.

Complementary exploratory analyses examined the HDMC first-order subfactors (online supplemental table 3) and additional physician characteristics, including working context (online supplemental tables 4a, 4b, 5a and 5b). Given the large number of comparisons, no post-hoc pairwise tests were conducted for the first-order subfactor analyses. Due to unequal representation and small sample sizes across specialty groups (see table 1), no inferential statistics were conducted for this variable. However, descriptive statistics on HDMC endorsement by medical specialty are available in online supplemental table 6.

Because interpretation of subgroup mean differences requires sufficient measurement equivalence across groups, measurement invariance was additionally examined for the four subgroup comparisons central to the study: gender (women vs men), age (<45 vs ≥45 years), physician type (GP vs specialist) and career status (resident vs attending physician). Initial analyses using the original 21-item hierarchical HDMC measurement model revealed estimation and convergence difficulties. Following methodological consultation and re-examination of the model specification, a revised 20-item model was used for the final measurement-invariance analyses, as described above and presented in online supplemental figure 3.

Configural, metric and scalar invariance were examined sequentially using multigroup structural equation modelling with continuous indicators and maximum-likelihood estimation. When full scalar invariance was not supported, partial scalar invariance was evaluated iteratively. At each step, item intercepts were examined using Stata’s score tests for group invariance; the intercept with the largest score-test statistic was freed across groups, and the model was re-estimated. This procedure was repeated until the changes in approximate fit indices relative to the metric model met the specified scalar-invariance criteria. Measurement invariance was considered supported when changes in the comparative fit index (ΔCFI), root mean square error of approximation (ΔRMSEA), and standardised root mean square residual (ΔSRMR) did not exceed 0.010, 0.015, and 0.030, respectively, for metric invariance, or 0.010 for ΔSRMR in scalar invariance. Because HDMC items are measured on Likert-type response scales, we also attempted an ordinal-indicator specification in Stata using generalised structural equation modelling (gsem, ordered logit). However, these ordinal models encountered convergence difficulties, preventing estimation of a stable sequence of configural, metric and scalar models. In addition, gsem did not provide the global fit indices (CFI, RMSEA and SRMR) required to apply the criteria used to evaluate changes in model fit across invariance levels. The continuous-indicator SEM specification was therefore retained for the final measurement-invariance analyses. Full results are reported in online supplemental table 8.

To address potential confounding effects and shared variance among physician characteristics, multivariable linear regression analyses were then performed. Separate models were constructed for each of the three HDMC dimensions. Physician characteristics were entered simultaneously using the enter method and categorical variables were dummy coded to facilitate the analysis of categorical differences. This allowed us to examine associations between physician characteristics and each HDMC dimension while accounting for other characteristics included in the model. Age and weekly working hours were modelled as continuous variables in the primary analyses. Regression coefficients are reported as unstandardised coefficients (*B*) with 95% CIs and standardised regression coefficients (β), and overall model fit was summarised using the *F* statistic, R² and adjusted R². Sensitivity analyses compared this continuous specification with the detailed categories used in the subgroup comparisons and with the binary categorisations used in the originally submitted analyses (<45 vs ≥45 years and ≤50 vs >50 hours/week, respectively). These alternative specifications yielded broadly consistent patterns and are reported in online supplemental table 7.

Regression assumptions (including independence of errors, multicollinearity, casewise diagnostics, homoscedasticity and normality of residuals) were verified following Field’s guidelines for regressions,^19^ as described in the online supplemental appendix 1. The regression models used complete cases for all included covariates. Of the 1002 participants with complete data on the 20 retained HDMC items, 940 (93.8%) had complete covariate data and were included in each of the three primary models. Given the limited proportion of missing covariate data, complete-case analysis was retained.

Given the predominance of participants residing in Belgium, the three multivariable models were additionally re-estimated among those residing in Belgium with complete covariate data (n=867); the direction and overall pattern of associations was substantively unchanged.

All statistical tests were two-sided, with p<0.05 used as a conventional reference threshold; given the exploratory nature and multiplicity of the analyses, individual p values were not interpreted as confirmatory evidence. Statistical analyses were conducted using IBM SPSS Statistics V.28.0,^20^ except for the measurement-invariance analyses, which were conducted using Stata V.19.5.^21^

## Results

### Participants

A total of 1292 physicians accessed the survey, of whom 1238 provided informed consent and 1002 provided complete data for all 20 HDMC items retained in the revised model and were included in the present study (figure 1). Missingness in sociodemographic and professional characteristics was limited and is reported in table 1. Participants represented a range of ages (with the largest age group being physicians under 35 years old, 39.7%) and 64.6% identified as women. The sample included attending GPs (27.7%), attending specialists (45.6%) and medical residents (24.9%). Most physicians worked 40 hours or more per week (84.4%) and practised primarily in hospital (specialists) or multidisciplinary settings (GPs). Specialists covered a broad spectrum of disciplines, with internal medicine, acute medicine, surgery, anaesthesiology and paediatrics being the most represented. Most participants resided in Belgium (n=922, 92.0%), while 65 (6.5%) resided in France, 2 (0.2%) in Switzerland and 6 (0.6%) in other countries; country of residence was missing for 7 participants (0.7%). Compared with 2023 Belgian workforce data for active French-speaking physicians, the Belgian subsample had a similar distribution of GPs and specialists (36.9% vs 35.7% GPs, respectively), but was younger (mean age 40.5 vs 51 years among GPs and 40.9 vs 49 years among specialists) and included a higher proportion of women (72.2% vs 49.9% among GPs and 62.3% vs 49.3% among specialists).^22^

**Figure 1:**
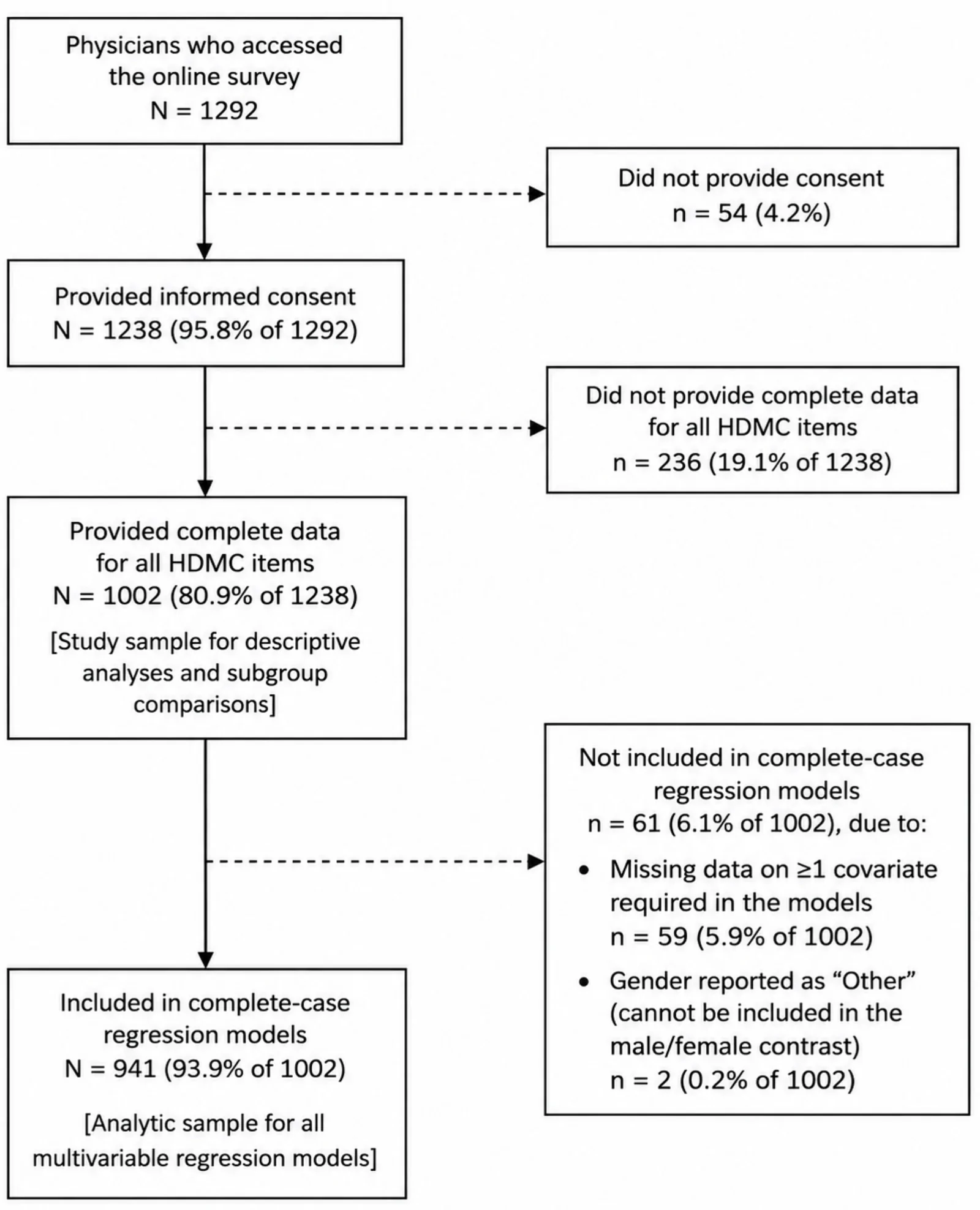
Flow diagram of participant inclusion. Flow diagram illustrating participant selection and inclusion in the present study. Percentages are reported relative to the preceding group unless otherwise specified. HDMC, harmful dimensions of medical culture.

### Descriptive statistics on the HDMC

Descriptive statistics for the three HDMC dimension scores, and for descriptive purposes only, the global HDMC score are presented in table 3. Cronbach’s alpha coefficients ranged from 0.61 to 0.85 across the three HDMC dimensions, with limited internal consistency for Stigma towards Burnout (α=0.61). Mean scores were 3.68 (SD=0.64) for Physicians’ Professional Commitment, 3.64 (SD=0.75) for the Myth of the Invulnerable Physician and 2.51 (SD=0.85) for Stigma towards Burnout. The global HDMC score was 3.49 (SD=0.54). Descriptive statistics for the five HDMC first-order subfactors are provided in online supplemental table 1.

**Table 3:** Descriptive statistics for the revised 20-item HDMC model

HDMC dimension	# of items	Mean	SD	α
Physicians’ Professional Commitment	11	3.68	0.64	0.85
The Myth of the Invulnerable Physician	6	3.64	0.75	0.77
Stigma towards Burnout	3	2.51	0.85	0.61
Global HDMC score	20	3.49	0.54	0.85

N=1002.

α, Cronbach’s alpha.

### Measurement invariance

Measurement-invariance analyses of the revised 20-item HDMC model supported configural and metric invariance across gender, age, career status and physician type. Full scalar invariance was not supported for any of the four comparisons. Partial scalar invariance was subsequently supported for all four comparisons after sequentially freeing selected item intercepts: one for gender, five for age, three for career status and three for physician type. Changes in CFI, RMSEA and SRMR between the metric and final partial scalar models were within the specified thresholds for all four comparisons. These findings indicate that subgroup comparisons should be interpreted in light of partial rather than full scalar invariance. Full results are reported in online supplemental table 8.

### Group comparisons

Independent samples t-tests and one-way ANOVA analyses indicated significant differences in HDMC dimension scores according to several physician characteristics (see table 2). Effect sizes were generally small to moderate. Online supplemental figures 1 and 2 present mean HDMC dimension scores with 95% CIs across the age and weekly working hours categories used in the one-way ANOVA analyses, respectively.

#### Gender

Two participants identified their gender as other. Given the extremely small sample size, they were excluded from gender group comparisons. Women reported higher Myth of the Invulnerable Physician scores than men (|d|=0.56, p<0.001). No statistically significant gender differences were observed for Physicians’ Professional Commitment (|d|=0.09, p=0.180) or Stigma towards Burnout (|d|=0.11, p=0.086).

#### Age category

Scores differed across age categories for the Myth of the Invulnerable Physician (η²=0.111, p<0.001) and Stigma towards Burnout (η²=0.040, p<0.001), but not for Physicians’ Professional Commitment (η²=0.004, p=0.391). Post-hoc comparisons generally indicated higher Myth of the Invulnerable Physician scores in younger than in older age groups, with the largest difference observed between physicians aged <35 and ≥65 years (mean difference=0.89, p<0.001). For Stigma towards Burnout, physicians aged <35 years reported higher scores than each of the older age groups (all p≤0.030). Full statistically significant post-hoc comparisons are reported in online supplemental table 2.

#### Relationship status

No statistically significant differences according to relationship status were observed for Physicians’ Professional Commitment (|d|=0.02, p=0.808), the Myth of the Invulnerable Physician (|d|=0.06, p=0.498) or Stigma towards Burnout (|d|=0.01, p=0.885).

#### Parental status

Physicians without children reported significantly higher scores on the Myth of the Invulnerable Physician (|d|=0.41, p<0.001) and Stigma towards Burnout (|d|=0.28, p<0.001) than physicians with children. No significant group differences were found for Physicians’ Professional Commitment (|d|=0.06, p=0.328).

#### Physician type

Specialist physicians reported higher Professional Commitment (|d|=0.49, p<0.001) and Stigma towards Burnout scores (|d|=0.25, p<0.001) than GPs. No statistically significant difference was observed for the Myth of the Invulnerable Physician (|d|=0.10, p=0.135).

#### Career status

Residents reported higher scores than attending physicians on the Myth of the Invulnerable Physician (|d|=0.42, p<0.001) and Stigma towards Burnout (|d|=0.31, p<0.001). No statistically significant difference was observed for Physicians’ Professional Commitment (|d|=0.06, p=0.456).

#### Weekly working hours

Scores differed across weekly working-hours categories for Physicians’ Professional Commitment (η²=0.104, p<0.001), the Myth of the Invulnerable Physician (η²=0.009, p=0.027) and Stigma towards Burnout (η²=0.018, p<0.001). Post-hoc comparisons indicated that physicians working >50 hours/week reported higher Professional Commitment scores than those in each of the other working-hours categories (all p<0.001). For Stigma towards Burnout, physicians working >50 hours/week reported higher scores than those working 30–39 hours/week (p=0.008) and 40–50 hours/week (p=0.002). No pairwise comparison for the Myth of the Invulnerable Physician remained statistically significant after adjustment. Full statistically significant post-hoc comparisons are reported in online supplemental table 2.

### Multivariable linear regressions

Results of the multivariable linear regressions are presented in table 4. Participants included in the complete-case regression analyses (n=940) did not differ from those not included (n=62) in age, gender (women vs men), relationship status, parental status, physician type, career status or HDMC dimension scores (all p>0.05). A small difference was observed for weekly working hours, with participants not included in the regression models reporting slightly fewer working hours (46.48 vs 49.82 hours/week; |d|=0.28, p=0.038).

**Table 4:** Multivariable linear regression analyses of associations between sociodemographic and professional characteristics and HDMC dimension scores

Physician characteristics	Physicians’ Professional Commitment			Myth of the Invulnerable Physician			Stigma towards Burnout		
	**B (95% CI**)	**β**	**P value**	**B (95% CI**)	**β**	**P value**	**B (95% CI**)	**β**	**P value**
Constant	2.60 (2.32 to 2.88)		<0.001	3.88 (3.55 to 4.21)		<0.001	2.33 (1.94 to 2.72)		<0.001
Age (years)	0.003 (−0.002 to 0.007)	0.05	0.232	−0.019 (−0.024 to −0.014)	−0.32	<0.001	−0.009 (−0.015 to −0.003)	−0.13	0.004
Gender: woman (ref: man)	0.14 (0.06 to 0.23)	0.11	<0.001	0.32 (0.22 to 0.42)	0.20	<0.001	0.08 (−0.04 to 0.20)	0.05	0.183
Relationship status: in a relationship (ref: single)	0.05 (−0.06 to 0.15)	0.03	0.411	0.07 (−0.05 to 0.19)	0.03	0.279	−0.02 (−0.16 to 0.13)	−0.01	0.801
Parental status: children (ref: no children)	−0.07 (−0.18 to 0.05)	−0.05	0.245	0.02 (−0.12 to 0.15)	0.01	0.821	0.00 (−0.15 to 0.16)	0.00	0.983
Physician type: specialist (ref: general practitioner)	0.20 (0.11 to 0.28)	0.15	<0.001	0.07 (−0.03 to 0.17)	0.04	0.171	0.15 (0.03 to 0.27)	0.08	0.014
Career status: resident (ref: attending physician)	−0.08 (−0.20 to 0.04)	−0.06	0.194	−0.09 (−0.23 to 0.05)	−0.05	0.203	0.09 (−0.07 to 0.26)	0.05	0.271
Weekly working hours	0.015 (0.012 to 0.019)	0.29	<0.001	0.005 (0.001 to 0.009)	0.08	0.013	0.008 (0.003 to 0.012)	0.11	0.001
Model fitting (*F*) R² Adjusted R²	*F*(7, 932)=19.84, p<0.001 0.13 0.12			*F*(7, 932)=24.74, p<0.001 0.16 0.15			*F*(7, 923)=8.66, p<0.001 0.06 0.05		

Age and weekly working hours were entered as continuous variables; their unstandardised coefficients represent the estimated difference in the outcome associated with a 1-year increase in age and a 1-hour/week increase in weekly working hours, respectively. For categorical variables, the reference categories are indicated directly in the table. All associated factors were entered simultaneously in each model. Models were estimated using complete cases for all included covariates. All p values are two-sided. B and its 95% CI are reported to three decimal places for age and weekly working hours because these continuous predictors have small per-unit coefficients that would be obscured by rounding to two decimal places; coefficients for categorical predictors are reported to two decimal places.

N=940 complete cases.

.B, unstandardised regression coefficient; HDMC, harmful dimensions of medical culture; β, standardised regression coefficient.

#### Physicians’ Professional Commitment

The model accounted for 13.0% of the variance in Physicians’ Professional Commitment scores (adjusted R²=0.12). Higher scores were associated with female gender, being a specialist rather than a GP and longer weekly working hours. No independent associations were observed for age, relationship status, parental status or career status.

#### Myth of the Invulnerable Physician

The model for the Myth of the Invulnerable Physician explained 15.7% of the variance in scores (adjusted R²=0.15). Higher scores were associated with younger age, female gender and longer weekly working hours. No independent associations were observed for relationship status, parental status, physician type, or career status.

#### Stigma towards Burnout

The model for Stigma towards Burnout had an adjusted R² of 0.05. Higher scores were associated with younger age, being a specialist rather than a GP and longer weekly working hours. No independent associations were observed for gender, relationship status, parental status or career status.

Overall, the models thus explained modest proportions of variance in HDMC dimension scores, with adjusted R² values ranging from 0.05 to 0.15.

Sensitivity analyses using binary and detailed categorical specifications of age and weekly working hours yielded broadly consistent patterns of associations with the primary continuous models (online supplemental table 7). In an additional sensitivity analysis restricted to participants residing in Belgium (n=867 complete cases), the results were consistent with those of the primary analyses, with no substantive changes in the direction or overall pattern of associations.

## Discussion

This study examined whether physicians’ endorsement of HDMC—namely Physicians’ Professional Commitment, the Myth of the Invulnerable Physician and Stigma towards Burnout—varied according to demographic and professional characteristics.

The findings revealed dimension-specific patterns rather than uniformly higher endorsement across particular physician groups. In the multivariable analyses, higher Physicians’ Professional Commitment scores were associated with female gender, specialist rather than general-practitioner status and longer weekly working hours. Higher Myth of the Invulnerable Physician scores were associated with younger age, female gender and longer weekly working hours, while higher Stigma towards Burnout scores were associated with younger age, specialist status and longer weekly working hours. After adjustment, relationship status, parental status and career status were not independently associated with any of the three dimensions, despite some differences by parental and career status in the unadjusted comparisons. Effect sizes in the unadjusted comparisons were generally small to moderate, with the largest differences observed for the Myth of the Invulnerable Physician across gender and age groups and for Physicians’ Professional Commitment across physician type and working-hours categories. The multivariable models explained modest proportions of variance, indicating that most variation in HDMC endorsement was not accounted for by the demographic and professional characteristics examined here. These findings suggest heterogeneity in the endorsement of specific medical cultural norms across physician groups, while cautioning against interpreting statistically significant differences as evidence of large or uniform cultural differences.

The observed associations can be considered in light of existing literature on professional socialisation and physician well-being, although the cross-sectional design precludes determining the mechanisms underlying these differences.

Younger age was associated with higher scores on the Myth of the Invulnerable Physician and Stigma towards Burnout, but not with Physicians’ Professional Commitment after adjustment. One possible contextual interpretation relates to professional socialisation during training and early career, when professional identity formation and exposure to the norms of medicine may be particularly salient.^13 23 24^ Role modelling, the hidden curriculum and implicit expectations surrounding resilience and sacrifice may contribute to the professional context in which such norms are encountered.^25–27^ This may coexist with an apparent generational shift towards greater openness around mental health, as discussed in recent literature.^28 29^ However, the present cross-sectional age differences cannot distinguish professional socialisation or ageing effects from cohort differences, career selection, or survivor effects.

Gender differences were also dimension-specific: women reported higher adjusted scores on Physicians’ Professional Commitment and the Myth of the Invulnerable Physician, but not on Stigma towards Burnout. These findings may be considered in the context of literature describing historically gendered professional expectations in medicine^30 31^ and persistent gender differences in caregiving and emotional labour.^32–35^ However, these contextual factors were not directly examined here and cannot be assumed to account for the observed associations. Importantly, higher Physicians’ Professional Commitment scores should not be interpreted as indicating that women, specialists or physicians working longer hours are simply more professionally committed. This HDMC dimension specifically captures work-prioritising, self-sacrificing and identity-central aspects of professional commitment; professional commitment itself is not inherently harmful.

Specialists reported higher adjusted scores than GPs on Physicians’ Professional Commitment and Stigma towards Burnout, but not on the Myth of the Invulnerable Physician. Although previous literature has described differences in professional cultures and norms across medical fields,^36 37^ the present comparison cannot establish that specialty-specific socialisation accounts for these differences. GPs and specialists may also differ in work setting, employment arrangements, workload and organisational culture, which may partly account for the observed associations. Finally, weekly working hours were the only characteristic associated with all three HDMC dimensions in the primary adjusted models, although the association with the Myth of the Invulnerable Physician was less robust across alternative specifications of working hours. Longer working hours may occur in professional environments in which dedication, stoicism and self-sacrifice are more strongly emphasised,^11 28 38 39^ but the direction of this relationship cannot be established: endorsement of such norms may also influence physicians’ willingness to accept or maintain longer working hours.

These findings have implications for how variation in medical cultural norms is conceptualised and addressed. Previous studies using the HDMC have reported associations between these cultural dimensions and physician burnout,^12^ as well as depressive symptoms and health-related behaviours.^14^ The present findings add that endorsement of specific HDMC dimensions may vary according to some demographic and professional characteristics, but do not establish whether these cultural norms contribute to differences in physician well-being or whether physician characteristics shape their endorsement. The generally small to moderate subgroup differences further argue against defining particular physician groups as uniformly vulnerable. Rather, efforts to understand and address medical culture should be attentive to variation across physicians and to the contexts in which norms surrounding sacrifice, invulnerability and help-seeking are expressed. Constructive role modelling, legitimising vulnerability and help-seeking and openly discussing physician distress have been proposed as potential avenues for cultural change, particularly within medical education.^40 41^ Such efforts should also consider structural conditions such as workload and organisational practices, which may interact with professional expectations.^11^

This study has several strengths, including a large and professionally diverse physician sample, the examination of multiple demographic and professional characteristics and the reporting of effect sizes alongside subgroup comparisons. Several limitations should nevertheless be considered. First, the cross-sectional design precludes temporal or causal inference and limits our ability to distinguish the mechanisms underlying the observed associations. Second, convenience and partly snowball-based recruitment may have introduced selection bias, for example, if physicians with greater interest in medical culture or physician well-being were more likely to participate. Although the Belgian subsample had a similar distribution of GPs and specialists to the population of active French-speaking physicians in Belgium, it was younger and included a higher proportion of women and should not be considered representative. The predominance of Belgian participants also limits generalisability to other contexts, although the Belgium-only sensitivity analyses yielded similar findings. Third, because participation was anonymous, no technical procedure was implemented to identify or prevent duplicate responses. Fourth, participants’ organisations were not collected to preserve anonymity, precluding adjustment for potential organisational clustering; unmeasured organisational characteristics may therefore partly account for variation in HDMC endorsement.

Psychometric considerations also warrant caution. The original HDMC was developed and psychometrically examined using the same dataset,^12^ and has not yet undergone independent evaluation in a new sample. Measurement-invariance analyses led to a post-hoc refinement of the measurement model and use of the revised 20-item model. Although configural and metric invariance were supported and partial scalar invariance was achieved across the four principal subgroup comparisons, full scalar invariance was *not* supported. The principal subgroup analyses compared observed composite scores, and partial scalar invariance does not ensure that these observed-score differences are entirely free from measurement-related influences. This is particularly relevant to the age comparison, for which five item intercepts had to be freed. Differences between groups should therefore be interpreted as exploratory patterns rather than unequivocal differences in the underlying latent constructs. Internal consistency was also limited for Stigma towards Burnout, and ordinal invariance models could not be stably estimated.

The revised model therefore requires independent evaluation of its factorial structure, measurement invariance, reliability and convergent, discriminant, and criterion-related validity.

Future research should examine how endorsement of HDMC dimensions varies across medical education and professional life using longitudinal designs capable of distinguishing ageing, cohort and career-stage effects and clarifying potential bidirectional relationships with characteristics such as workload. Studies in more systematically recruited and diverse physician samples should also examine the organisational and structural factors that may account for variation in these norms and their interplay with individual and professional characteristics. Other unmeasured characteristics, including potentially relevant factors such as self-valuation^17^ and imposter phenomenon^42^ may also contribute to variation in HDMC endorsement. Ultimately, a better understanding of how professional norms are expressed, maintained and potentially changed across medical contexts may help inform approaches that reconcile physician well-being with professional commitment and patient care.

### Conclusions

Physicians’ endorsement of harmful dimensions of medical culture varied across demographic and professional characteristics, with distinct patterns across the three HDMC dimensions. Age, gender, physician type and weekly working hours showed different patterns of association across these dimensions rather than uniformly higher endorsement within particular physician groups. Although the observed associations were generally modest and subgroup comparisons require cautious interpretation, the findings highlight heterogeneity in how specific medical cultural norms are endorsed across physicians. Further research in independent and diverse samples is needed to examine how individual, professional, organisational and cultural contexts may shape these patterns.

## Supplementary material

10.1136/bmjopen-2026-124359 online supplemental file 1

## Data Availability

Data are available in a public, open access repository.
