# Supplementary material for: Harmful dimensions of medical culture across physician subgroups: a cross-sectional study of 1002 physicians predominantly practising in Belgium
Source: BMJ Open. 2026 Oct 20;16(10):e124359. doi: 10.1136/bmjopen-2026-124359 (PMC13684193; doi:10.1136/bmjopen-2026-124359)
Supplement: Supplementary file 1 [file bmjopen-2026-124359.reviewer_comments.pdf]

PEER REVIEW HISTORY

BMJ Open publishes all reviews undertaken for accepted manuscripts. Reviewers are asked to complete a checklist review form and are provided with free text boxes to elaborate on their assessment. These free text comments are reproduced below.

ARTICLE DETAILS

Title (Provisional)

Harmful dimensions of medical culture across physician subgroups: a cross-sectional study of 1002 physicians predominantly practising in Belgium

Authors

Banse, Emilie; Mikolajczak, Moïra; Bayot, Marie; Lenoir, Anne-Laure; De Timary, Philippe

---

VERSION 1 - REVIEW

Reviewer 1

Name Son, Daisuke

Affiliation Tottori University, Department of Community-based Family Medicine, Faculty of Medicine

Date 10-Jul-2026

COI None

This manuscript addresses an important and underexamined topic: whether endorsement of potentially harmful professional norms differs across physician subgroups. The large and professionally diverse sample, the transparent acknowledgement that the dataset has been used in previous publications, and the multidimensional assessment of medical culture are strengths.

However, I have substantial concerns regarding the internal consistency of the reported numerical results, the validity of comparisons across subgroups, the handling of continuous and missing data, and the interpretation of cross-sectional associations. In its current form, the manuscript requires a comprehensive statistical and reporting audit before the findings can be reliably evaluated.

Major comments

1. Numerous numerical inconsistencies require a complete audit of the dataset, analyses, and tables

There are multiple discrepancies within and across Tables 1–4 and the supplementary materials. These are not merely cosmetic because several affect the principal findings.

Examples include:

In Table 1, the number of female participants is reported as 547 (64.6%). However, 64.6% of 1002 is approximately 647, and Table 3 reports 647 female participants.

In Table 1, the percentages for parental status appear to be reversed: 415 participants without children are reported as 58.1%, whereas 582 participants with children are reported as 41.4%.

The specialty section is labelled N=645, but the listed specialty counts sum to 631; adding 18 missing cases produces 649 rather than 645. In addition, 25 participants in gynaecology/obstetrics cannot represent 3.4% of either 631 or 645.

In Table 3, the global HDMC means across age categories appear to have been displaced across rows. The supplementary post-hoc differences imply an approximately monotonic sequence of 3.51, 3.44, 3.41, 3.35, and 3.21, rather than the sequence currently displayed.

The Professional Commitment mean for physicians working 40–50 hours is reported as 3.39. This is inconsistent with the overall mean and with the post-hoc comparisons; 3.59 appears more plausible, but this must be verified from the source data.

In Supplementary Table 2, the Professional Commitment difference of −0.58 labelled "<30 h vs 40–50 h" is incompatible with the means and likely refers to "<30 h vs >50 h."

The reported Stigma towards Burnout difference for "40–50 h vs >50 h" should have a negative rather than positive sign based on the displayed means.

In Table 4, the coefficient for physician status in the global HDMC model is −0.06, with a reported 95% CI of −0.16 to 0.30 and p=0.180. This confidence interval is incompatible with both the coefficient and p value; the upper bound may be 0.03.

The text states that residents differed from attending physicians only on the Myth of the Invulnerable Physician and the global score. Table 3 also shows a significant difference in Stigma towards Burnout (p<0.001).

I strongly recommend regenerating all descriptive statistics, tables, figures, confidence intervals, and narrative results directly from a single reproducible statistical script. An independent author or statistician should verify them against the raw data. All corrections should then be propagated to the abstract, Results, Discussion, and supplementary material.

2. Measurement invariance is essential for the central subgroup comparisons

The main objective is to compare HDMC scores across gender, age, specialty, and career-stage groups. Such comparisons assume that the scale measures the same constructs in the same way across these groups. However, no measurement-invariance or differential-item-functioning analysis is reported.

This is particularly important because the scale was developed and psychometrically evaluated by the same research group using this same dataset. Apparent group differences

could reflect differences in item interpretation, thresholds, or factor structure rather than genuine differences in the latent constructs.

Please consider testing, where sample sizes permit, configural, metric, and scalar invariance across at least the principal comparisons, such as gender, younger versus older physicians, general practitioners versus specialists, and residents versus attending physicians. For ordinal Likert items, an ordinal CFA or appropriate differential-item-functioning approach would be preferable. If scalar invariance cannot be established, direct comparisons of observed means should be interpreted much more cautiously.

The manuscript should also explicitly acknowledge that independent external validation of the HDMC scale has not yet been demonstrated.

3. The calculation and interpretation of the global HDMC score are unclear

The Methods state that the global score is calculated as the mean of the three dimension scores. However, the overall dimension means of 3.68, 3.64, and 2.56 would produce a mean of approximately 3.29, not the reported global mean of 3.43. An item-weighted mean based on 11, 6, and 4 items would also not exactly reproduce the reported value.

Please provide the precise scoring algorithm, including:

whether dimensions, first-order subfactors, or individual items are equally weighted;

whether any items are reverse scored;

how the global score differs from a simple mean of all 21 items;

how missing item responses were handled; and

whether this exact scoring procedure was prespecified and validated in the previous psychometric study.

The global score is central to the abstract and conclusions, so its calculation must be fully reproducible.

The description of Cronbach's alpha values of 0.62–0.87 as "acceptable to very good" is also somewhat optimistic. An alpha of 0.62 indicates limited internal consistency and should be described more cautiously. Reporting omega coefficients may be informative, particularly given the multidimensional structure of the scale.

4. Dichotomising age and working hours is insufficiently justified and may distort the associations

Age and working hours appear to have been collected as continuous variables but were first categorised for group comparisons and then dichotomised for regression. The stated rationale—that dichotomisation maintains parsimony and limits multicollinearity—is not statistically convincing. Dichotomisation generally reduces information and statistical power, creates arbitrary thresholds, and may leave residual confounding; it does not inherently solve multicollinearity.

Please consider modelling age and working hours continuously. Potential non-linearity can be assessed using restricted cubic splines, polynomial terms, or clearly justified categories. At minimum, the authors should provide sensitivity analyses comparing continuous, categorical, and binary specifications.

The coding also requires clarification. The table legend identifies “<50 hours” as the reference group while the comparison group is “>50 hours,” leaving exactly 50 hours conceptually unclear. Presumably the intended reference is ≤50 hours.

Regression rows should identify the actual contrast, for example, “Age ≥45 years, reference <45 years,” rather than simply “Age.” This would also make the negative coefficients in the abstract easier to interpret.

5. Participant flow, missing data, recruitment, and the regression sample require clearer reporting

The manuscript states that 1002 physicians “provided complete data for all survey items,” yet Table 1 reports missing data for several covariates. Furthermore, the regression degrees of freedom indicate an analytic sample of approximately 948 participants, not 1002.

Please:

distinguish completeness of HDMC items from completeness of all study variables;

report the exact sample size used in every regression model;

quantify separately the reasons for exclusion among the 1293 people who accessed the survey;

state how many were excluded because of ineligibility, incomplete HDMC data, or missing covariates;

describe the amount and pattern of missingness for each predictor;

consider a multiple-imputation or complete-case sensitivity analysis; and

compare included and excluded participants where feasible.

The flow diagram currently provides no numerical breakdown for the 291 excluded respondents and refers vaguely to “the exact analytic sample.” It should show explicit counts at every stage.

The manuscript should also report:

the recruitment dates;

the exact numbers recruited from Belgium and France;

whether country was included in, or tested as, a covariate;

whether duplicate anonymous responses were prevented;

a study-size or precision rationale; and

the extent to which the achieved sample resembles the target physician population.

The phrase “a convenience sample representative of the broad physician population” should be revised. A convenience sample may be diverse, but representativeness cannot be assumed without a sampling frame and population comparison.

Because recruitment included France as well as Belgium, a sensitivity analysis restricted to Belgian participants, or adjustment for country, would be useful. National differences in training, employment structures, and professional culture could influence HDMC scores.

6. Multiplicity and the exploratory nature of the analyses need to be addressed

A large number of comparisons were performed across four principal outcomes, numerous physician characteristics, seven subfactors, professional settings, and specialties. Bonferroni adjustment was applied to selected post-hoc comparisons, but there appears to be no correction or explicit multiplicity strategy for the broader set of tests.

Please clearly identify which outcomes and comparisons were primary, secondary, or exploratory. Either an appropriate multiplicity approach, such as false-discovery-rate control, should be considered, or the analyses should be consistently presented as exploratory, with emphasis on estimates, confidence intervals, and effect sizes rather than binary statistical significance.

The OSF registration is described as registration of the broader research project. Please state explicitly whether the research question, subgroup analyses, variable dichotomisations, and regression models in the present manuscript were preregistered before examining the data. If not, they should be labelled as post-hoc exploratory analyses.

As this is at least the third publication using the same dataset, the authors should provide a concise statement specifying the distinct aims, variables, and analyses of each publication and explaining how duplicate reporting has been avoided.

7. Effect sizes and practical importance should receive more attention

With approximately 1000 participants, relatively small differences can achieve very low p values. The manuscript should report effect sizes for the unadjusted analyses, such as Cohen’s d and η^2 or ω^2, and preferably standardised coefficients or partial R^2 for regression terms.

For example, a global-score difference of approximately 0.15–0.20 points on a 1–5 scale may be statistically robust but modest in practical magnitude. The Discussion should distinguish statistical significance from meaningful cultural or clinical differences. The relatively low adjusted R^2 values of 0.04–0.12 also indicate that most variation in HDMC endorsement remains unexplained.

8. Several conclusions exceed what can be inferred from this cross-sectional analysis

Terms including “antecedents,” “risk factors,” “predictors,” “vulnerable,” and “contribute to” imply temporal or causal relationships that are not established here. These should generally be replaced by “associated factors,” “correlates,” or “groups reporting higher scores.”

In particular:

Long working hours may reinforce self-sacrificing norms, but physicians who endorse such norms may also choose or accept longer hours.

Cross-sectional age differences cannot distinguish ageing effects, cohort effects, career selection, or survivor effects.

The general practitioner–specialist contrast may reflect work setting, employment arrangements, workload, or organisational culture rather than specialty-specific socialisation.

The finding that younger physicians score higher on some dimensions does not establish that they are more likely to “internalise” norms during training.

“Strong endorsement” should not be used unless validated thresholds or normative categories exist.

The conclusions also group younger physicians, female physicians, specialists, and physicians with long working hours together as endorsing norms of self-sacrifice, invulnerability, and burnout stigma. The actual associations are dimension-specific. For example, gender is principally associated with Professional Commitment and the Myth of the Invulnerable Physician, whereas age is associated with invulnerability and stigma. The abstract and Discussion should map each characteristic to the relevant outcome rather than implying that all groups scored higher on all dimensions.

9. The conceptual meaning of “Physicians’ Professional Commitment” needs greater nuance

Professional commitment can be adaptive and central to good patient care. The measured dimension appears to combine work–life interference, sacrifice, and centrality of professional identity. The authors should clarify why a high score is interpreted as harmful and distinguish healthy professional commitment from maladaptive overcommitment or self-sacrificing commitment.

Otherwise, describing physicians with high professional commitment as endorsing a “harmful dimension” may inadvertently pathologise professional dedication. A more precise dimension label may be warranted.

Minor comments

The Results heading “Hierarchical regressions” is inconsistent with the Methods, which describe simultaneous entry using the enter method. These are multivariable linear regressions, not hierarchical regressions.

The Table 3 legend states that “correlation is significant.” These are group-comparison tests, not correlations.

Please specify which analyses used Welch’s ANOVA. If homogeneity of variance was violated, clarify which post-hoc procedure was used; standard equal-variance pairwise procedures may not be appropriate, and Games–Howell comparisons may be preferable.

The supplementary mean plots should include uncertainty intervals. Point-and-error-bar plots would be more informative than connected lines, which may imply continuous trajectories across arbitrary categories.

“Most physicians worked over 40 hours” should be “40 hours or more” if the category includes exactly 40 hours.

Please use “gender” and “sex” consistently and explain exactly how the variable was assessed. The Discussion currently refers to “female sex,” whereas the variable is labelled gender.

“Trial registration” is not the most appropriate label for an observational survey. “Study preregistration” would be clearer.

The keyword “risk factors” should be replaced with “associated factors” or “physician characteristics.”

The abstract’s description of “primary and secondary outcome measures” does not distinguish primary from secondary outcomes.

In the Acknowledgements, “participated to the online survey” should be “participated in the online survey.” Because two individuals are acknowledged, “for her help” should be “for their help.”

Specialty terminology should be reviewed, for example, “obstetrics and gynaecology” rather than “gynaecology and obstetrical.”

The STROBE checklist appears to state that interactions and a study-size explanation are reported, although these are not evident in the manuscript. The checklist should be corrected.

**Reviewer**	**2**
**Name**	**Mete, Mihriye**
**Affiliation**	**MedStar Health Research Institute, CBIDS**
**Date**	**28-Jul-2026**
**COI**	**None**

The empirical exploration of the culture in which physicians operate is a significant contribution to the literature. The paper is very well written. Statistical analyses are appropriate. Please clarify the following:

--hierarchical regressions are mentioned in the results but the stats plan section does not explain this approach. The analyses can be redone with using linear mixed methods with random effects at the organizational level. Given the snowball nature of the survey design/data collection, it becomes even more important to control for unexplained factors at the organizational level that are strongly correlated with culture--potentially.

--While the new scale is theoretically meaningful, overall results (including in the primary publication showing correlations with burnout) suggest that it is not strongly related to burnout (weak correlations with burnout) and that the effect sizes for the different categories of the physicians in relation to the subscales are practically negligible. These results lead to questions about the validity of the scale, especially construct validity.

--Given the nature of the survey design, selection bias is a serious problem that endangers the validity of the inference. This can't be addressed here but the authors might want to consider reaching out to other physician wellbeing researchers to further validate their scale in larger and more systematically collected samples (Stanford group for instance).

VERSION 1 - AUTHOR RESPONSE

Reviewer: 1 Dr. Daisuke Son, Tottori University

Comments to the Author: This manuscript addresses an important and underexamined topic: whether endorsement of potentially harmful professional norms differs across physician subgroups. The large and professionally diverse sample, the transparent acknowledgement that the dataset has been used in previous publications, and the multidimensional assessment of medical culture are strengths. However, I have substantial concerns regarding the internal consistency of the reported numerical results, the validity of comparisons across subgroups, the handling of continuous and missing data, and the interpretation of cross-sectional associations. In its current form, the manuscript requires a comprehensive statistical and reporting audit before the findings can be reliably evaluated.

Response: We thank the reviewer for this careful and constructive assessment. In response to these concerns, we conducted a comprehensive audit of the data, statistical analyses, tables, and reporting, and revised the manuscript accordingly. We also performed additional analyses to address the methodological issues raised. The specific changes and their implications are detailed in our point-by-point responses below.

1. Major comments

Numerous numerical inconsistencies require a complete audit of the dataset, analyses, and tables. There are multiple discrepancies within and across Tables 1-4 and the supplementary materials. These are not merely cosmetic because several affect the principal findings. Examples include:

- **In Table 1, the number of female participants is reported as 547 (64.6%). However, 64.6% of 1002 is approximately 647, and Table 3 reports 647 female participants.**

Response: Thank you for identifying this inconsistency. The correct number of women is 647 (64.6%), as reported in Table 3. The value of 547 in Table 1 was a typographical error and has been corrected accordingly.

- **In Table 1, the percentages for parental status appear to be reversed: 415 participants without children are reported as 58.1%, whereas 582 participants with children are reported as 41.4%.**

Response: Thank you for noting this error. The percentages for parental status were inadvertently reversed in Table 1. The correct values are 582 participants with children (58.1%) and 415 participants without children (41.4%); 5 participants (0.5%) had missing data for this variable. We have corrected Table 1 accordingly and verified these figures against the final dataset.

- **The specialty section is labelled N=645, but the listed specialty counts sum to 631; adding 18 missing cases produces 649 rather than 645. In addition, 25 participants in gynaecology/ obstetrics cannot represent 3.4% of either 631 or 645.**

Response: Thank you for identifying these inconsistencies. We re-audited the specialty variables against the final dataset and corrected the counts and percentages in Table 1 and the supplemental material. The physician-type variable identified 645 participants as specialists, of whom detailed medical-specialty information was available for 627. Among these 627 participants, 25 (4.0%) were in obstetrics and gynaecology. Online supplemental table 6 has been regenerated using this denominator.

- **In Table 3, the global HDMC means across age categories appear to have been displaced across rows. The supplementary post-hoc differences imply an approximately monotonic sequence of 3.51, 3.44, 3.41, 3.35, and 3.21, rather than the sequence currently displayed.**

Response: Thank you for identifying this error. The reviewer was correct that the global HDMC means across age categories had been displaced across rows. As detailed in our response to Comment 3, the global HDMC composite has subsequently been removed from the inferential subgroup analyses following revision of the measurement model and reconsideration of its inferential role. It is therefore no longer reported in Table 3.

- **The Professional Commitment mean for physicians working 40-50 hours is reported as 3.39. This is inconsistent with the overall mean and with the post-hoc comparisons; 3.59 appears more plausible, but this must be verified from the source data.**

Response: Thank you for identifying this error. We verified the Professional Commitment means directly against the final dataset. The correct mean for physicians working 40-50 hours per week is 3.59, rather than 3.39 as previously reported. Table 3 has been corrected accordingly.

- **In Supplementary Table 2, the Professional Commitment difference of -0.58 labelled "<30 h vs 40–50 h" is incompatible with the means and likely refers to "<30 h vs >50 h."**

Response: Thank you for identifying this error. We re-ran the post-hoc comparisons and confirmed that the mean difference of -0.58 corresponds to the comparison between physicians working <30 hours and >50 hours per week, rather than between <30 and 40-50 hours. The <30 vs 40-50 hours comparison was not statistically significant and has therefore been removed, as online supplemental table 2 reports only significant post-hoc comparisons. The significant <30 vs >50 hours comparison (mean difference = -0.58) has been added to the corrected table.

- **The reported Stigma towards Burnout difference for "40–50 h vs >50 h" should have a negative rather than positive sign based on the displayed means.**

Response: Thank you for identifying this inconsistency. Following revision of the Stigma towards Burnout dimension, we re-ran the subgroup and post-hoc analyses using the final three-item score. Online supplemental table 2 has been regenerated, and the direction and sign of all reported mean differences were verified against the corresponding group means.

- **In Table 4, the coefficient for physician status in the global HDMC model is −0.06, with a reported 95% CI of −0.16 to 0.30 and p=0.180. This confidence interval is incompatible with both the coefficient and p value; the upper bound may be 0.03.**

Response: Thank you for identifying this error. We verified the originally reported coefficient and confidence interval during the statistical audit. As detailed in our response to Comment 3, the global HDMC composite has since been removed from the inferential analyses; the revised Table 4 therefore reports multivariable models for the three HDMC dimensions only.

- **The text states that residents differed from attending physicians only on the Myth of the Invulnerable Physician and the global score. Table 3 also shows a significant difference in Stigma towards Burnout (p<0.001).**

Response: Thank you for identifying this omission. We have corrected the Results to report the complete comparison between attending physicians and residents across the three HDMC dimensions. In the revised analyses, residents reported higher scores than attending physicians on the Myth of the Invulnerable Physician (3.88 ± 0.61 vs 3.57 ± 0.78; p<0.001; d=0.42) and Stigma towards Burnout (2.76 ± 0.85 vs 2.42 ± 0.83; p<0.001; d=0.39), whereas no statistically significant difference was observed for Physicians' Professional Commitment (3.71 ± 0.64 vs 3.67 ± 0.64; p=0.448; d=0.06). Table 3 and the corresponding Results text have been revised accordingly.

I strongly recommend regenerating all descriptive statistics, tables, figures, confidence intervals, and narrative results directly from a single reproducible statistical script. An independent author or statistician should verify them against the raw data. All corrections should then be propagated to the abstract, Results, Discussion, and supplementary material.

Response: We agree that a comprehensive numerical audit was necessary. We returned to the final analysis dataset and used a reproducible statistical syntax to regenerate and verify sample counts and missing data, HDMC scoring, descriptive and subgroup analyses, multivariable regression models, and

supplementary analyses. Following revision of the measurement model, inferential analyses were restricted to the three HDMC dimensions, with the global composite retained for descriptive purposes only. All corrections were propagated to the tables, online supplemental material, Abstract, Results, and Discussion. Revised analyses were conducted using reproducible SPSS syntax, with separate Stata syntax for the measurement-invariance analyses. The statistical syntaxes have also been made available in the OSF repository, as indicated in the revised Data sharing statement.

2. Measurement invariance is essential for the central subgroup comparisons

The main objective is to compare HDMC scores across gender, age, specialty, and career-stage groups. Such comparisons assume that the scale measures the same constructs in the same way across these groups. However, no measurement-invariance or differential-item-functioning analysis is reported. This is particularly important because the scale was developed and psychometrically evaluated by the same research group using this same dataset. Apparent group differences could reflect differences in item interpretation, thresholds, or factor structure rather than genuine differences in the latent constructs. Please consider testing, where sample sizes permit, configural, metric, and scalar invariance across at least the principal comparisons, such as gender, younger versus older physicians, general practitioners versus specialists, and residents versus attending physicians. For ordinal Likert items, an ordinal CFA or appropriate differential-item-functioning approach would be preferable. If scalar invariance cannot be established, direct comparisons of observed means should be interpreted much more cautiously. The manuscript should also explicitly acknowledge that independent external validation of the HDMC scale has not yet been demonstrated.

Response: Thank you for this important comment. We agree that measurement invariance is important for interpreting observed mean differences across physician subgroups. We therefore conducted additional measurement-invariance analyses across the four principal comparisons: gender (women vs men), age (<45 vs ≥45 years), physician type (general practitioner vs specialist), and career status (resident vs attending physician).

Initial analyses using the original 21-item hierarchical HDMC measurement model revealed substantial estimation and convergence difficulties across subgroup analyses. We therefore sought methodological input from our university statistical consultation service and re-examined the higher-order model specification. Estimation difficulties persisted after applying appropriate identification constraints and appeared to be concentrated in the Stigma towards Burnout dimension, particularly item ec_10.

We therefore examined a simplified specification in which ec_10 was removed and Stigma towards Burnout was represented directly by its three remaining indicators (ec_5, ec_6, and ec_9), rather than through the previous two first-order factor structure. The structures of Physicians' Professional Commitment and the Myth of the Invulnerable Physician were unchanged. The resulting revised 20-item model could be successfully estimated across all four subgroup comparisons and was used for the subsequent measurement-invariance analyses. This refinement was undertaken post hoc in response to the measurement-invariance analyses requested during peer review. To maintain consistency, all analyses involving Stigma towards Burnout and the descriptive global HDMC composite were rerun using this final 20-item specification.

Using the revised model, we sequentially examined configural, metric, scalar, and, where required, partial scalar invariance using continuous-indicator SEM. Changes in approximate fit indices were evaluated using ΔCFI≤.010, ΔRMSEA≤.015, and ΔSRMR≤.030 for metric invariance and ≤.010 for scalar invariance. Configural and metric invariance were supported across all four subgroup comparisons. Full scalar invariance was not supported for any comparison. Partial scalar invariance was subsequently achieved after freeing one item intercept for gender, five for age, three for physician type, and three for career status. Changes in CFI, RMSEA, and SRMR between the metric and final partial scalar models were within the specified thresholds in all four comparisons. Full results are reported in online supplemental table 8.

Because the HDMC items are Likert-type indicators, we also attempted ordinal measurement-invariance analyses using gsem in Stata. These models encountered convergence difficulties and did not yield a stable sequence of configural, metric, and scalar models. In addition, gsem did not provide the global fit indices (CFI, RMSEA, and SRMR) required to apply the criteria used in the continuous-indicator analyses. We therefore retained the continuous-indicator SEM approach and now acknowledge the inability to establish ordinal invariance as a methodological limitation.

These analyses therefore support partial, but not full, scalar invariance of the revised 20-item HDMC model across the four principal subgroup comparisons. We have added the measurement-invariance procedures and results to the Methods and Results, with detailed estimates in online supplemental table 8 and the revised measurement model in online supplemental figure 3. Subgroup differences are now interpreted cautiously in light of partial rather than full scalar invariance, and the Discussion explicitly acknowledges that the revised 20-item model requires independent psychometric evaluation in new samples.

3. The calculation and interpretation of the global HDMC score are unclear

The Methods state that the global score is calculated as the mean of the three dimension scores. However, the overall dimension means of 3.68, 3.64, and 2.56 would produce a mean of approximately 3.29, not the reported global mean of 3.43. An item-weighted mean based on 11, 6, and 4 items would also not exactly reproduce the reported value. Please provide the precise scoring algorithm, including:

- **whether dimensions, first-order subfactors, or individual items are equally weighted;**
- **whether any items are reverse scored;**
- **how the global score differs from a simple mean of all 21 items;**
- **how missing item responses were handled; and**
- **whether this exact scoring procedure was prespecified and validated in the previous psychometric study.**

The global score is central to the abstract and conclusions, so its calculation must be fully reproducible.

Response: Thank you for drawing our attention to this discrepancy. We conducted a complete audit of the HDMC scoring procedure and agree that the calculation and role of the global score required clarification.

In the original psychometric study, the HDMC comprised 21 items distributed across three dimensions: 11 items for Physicians' Professional Commitment, 6 for the Myth of the Invulnerable Physician, and 4 for Stigma towards Burnout. A global score was calculated as the mean of all 21 individual items, rather than as the unweighted mean of the three dimension scores. Thus, individual items were equally weighted, dimensions and first-order subfactors were not equally weighted as separate units, and no items were reverse scored.

Following the measurement-model refinement described in our response to Comment 2, one Stigma towards Burnout item was removed. The revised descriptive global HDMC composite is therefore the mean of the 20 retained items and can equivalently be expressed as:

Global HDMC = (11 × Physicians' Professional Commitment + 6 × Myth of the Invulnerable Physician + 3 × Stigma towards Burnout) / 20.

All 1002 participants had complete responses to the 20 retained HDMC items, so no procedure for handling missing HDMC item responses was required. The revised global composite has a mean of 3.49 (SD=0.54). Because this 20-item composite results from the post-hoc measurement-model refinement undertaken during peer review, it was not the scoring specification examined in the original psychometric study.

The discrepancy identified by the reviewer resulted from a scoring error in the global composite reported in the original submission, in which one item had inadvertently been omitted from its calculation. This error affected only the global composite and did not affect any of the three HDMC dimension scores. Our re-examination also led us to reconsider the inferential role of this global score. The global HDMC score was a descriptive composite created by averaging the individual HDMC items; it was not specified as an overarching latent factor in the measurement model established in the original psychometric study, which instead comprised three distinct second-order dimensions. We therefore removed the global composite from the subgroup comparisons and multivariable regression analyses and now retain it for descriptive purposes only.

The Methods have been revised to provide the precise scoring procedure and to distinguish the original 21-item scoring from the revised 20-item descriptive composite. The Abstract, Results, tables, and online supplemental material have also been revised accordingly so that all inferential analyses and their interpretation are based on the three HDMC dimensions.

The description of Cronbach's alpha values of 0.62 - 0.87 as "acceptable to very good" is also somewhat optimistic. An alpha of 0.62 indicates limited internal consistency and should be described more cautiously. Reporting omega coefficients may be informative, particularly given the multidimensional structure of the scale.

Response: Thank you for this comment. We agree that the previous description of the Cronbach's alpha coefficients as "acceptable to very good" was overly positive. Following the revision of the HDMC measurement model described above, Cronbach's alpha coefficients for the three dimensions ranged from 0.61 to 0.85, with limited internal consistency for Stigma towards Burnout (α=0.61). We have revised the manuscript to report and interpret these coefficients more cautiously. We also considered the suggestion to report omega coefficients. Because the present study was not designed

as a further psychometric validation of the HDMC, we retained Cronbach's alpha for consistency with the original psychometric study. We now explicitly acknowledge, however, that the reliability and broader psychometric properties of the revised 20-item model (including alternative reliability estimates such as omega) require evaluation in independent samples.

4. Dichotomising age and working hours is insufficiently justified and may distort the associations

Age and working hours appear to have been collected as continuous variables but were first categorised for group comparisons and then dichotomised for regression. The stated rationale—that dichotomisation maintains parsimony and limits multicollinearity—is not statistically convincing. Dichotomisation generally reduces information and statistical power, creates arbitrary thresholds, and may leave residual confounding; it does not inherently solve multicollinearity. Please consider modelling age and working hours continuously. Potential non-linearity can be assessed using restricted cubic splines, polynomial terms, or clearly justified categories. At minimum, the authors should provide sensitivity analyses comparing continuous, categorical, and binary specifications.

Response: Thank you for this important comment. We agree that our original justification for dichotomising age and weekly working hours, particularly the reference to multicollinearity, was insufficient. We therefore re-ran the multivariable linear regression models for each of the three HDMC dimensions using three alternative specifications of age and weekly working hours: (1) as continuous variables, (2) using the detailed categories employed in the subgroup analyses, and (3) using the original binary variables.

The overall pattern of findings was broadly consistent across specifications. Age was not associated with Physicians' Professional Commitment but was inversely associated with the Myth of the Invulnerable Physician and Stigma towards Burnout. Greater weekly working hours were consistently associated with higher Physicians' Professional Commitment scores and generally with higher Stigma towards Burnout scores. The association between weekly working hours and the Myth of the Invulnerable Physician was less robust and was evident only in the continuous specification.

Because modelling age and weekly working hours continuously preserves the information contained in the original variables, avoids arbitrary cut-points, and provides a parsimonious specification, while yielding findings broadly consistent with the alternative specifications, we revised the primary multivariable analyses of the three HDMC dimensions to model both variables continuously. The binary and detailed categorical specifications are presented as sensitivity analyses in online supplemental table 7. We have revised the Statistical analyses section, Results, Table 4, Abstract, and corresponding interpretation of the findings accordingly.

The coding also requires clarification. The table legend identifies "<50 hours" as the reference group while the comparison group is ">50 hours," leaving exactly 50 hours conceptually unclear. Presumably the intended reference is ≤50 hours. Regression rows should identify the actual contrast, for example, "Age ≥45 years, reference <45 years," rather than simply "Age." This would also make the negative coefficients in the abstract easier to interpret.

Response: Thank you for these helpful comments. As described above, age and weekly working hours are now modelled continuously in the primary multivariable regression analyses and are therefore reported as continuous terms in the revised Table 4. The original binary specifications are retained only as sensitivity analyses in online supplemental table 7. In these analyses, we have clarified that the weekly working-hours contrast is >50 h/week versus ≤50 h/week, such that physicians reporting exactly 50 hours are included in the reference category. We have also explicitly identified the contrasts and reference categories for all categorical predictors. For example, the binary sensitivity analyses report "Age ≥45 years (ref: <45 years)" and "Weekly working hours >50 h/week (ref: ≤50 h/week)". The Results and Abstract have been revised accordingly so that the direction of associations is unambiguous.

5. Participant flow, missing data, recruitment, and the regression sample require clearer reporting

The manuscript states that 1002 physicians "provided complete data for all survey items," yet Table 1 reports missing data for several covariates. Furthermore, the regression degrees of freedom indicate an analytic sample of approximately 948 participants, not 1002.

Please:

- **distinguish completeness of HDMC items from completeness of all study variables;**
- **report the exact sample size used in every regression model;**
- **quantify separately the reasons for exclusion among the 1293 people who accessed the survey; state how many were excluded because of ineligibility, incomplete HDMC data, or missing covariates;**
- **describe the amount and pattern of missingness for each predictor;**
- **consider a multiple-imputation or complete-case sensitivity analysis; and**
- **compare included and excluded participants where feasible.**

Response: Thank you for highlighting this ambiguity and for these helpful suggestions. We have revised the manuscript to distinguish completeness of the HDMC items from completeness of other study variables and have clarified participant flow, covariate missingness, and the analytic sample used in the multivariable models.

Of the 1292 physicians who accessed the survey, 1238 provided informed consent and 1002 had complete data for all 20 HDMC items retained in the revised measurement model, constituting the study sample. Thus, 54 individuals did not provide consent, and 236 consenting participants did not have complete HDMC item data. There was no other exclusion for ineligibility. These counts are now reported explicitly in the revised participant flow diagram.

Among the 1002 participants in the study sample, missingness in the covariates included in the multivariable models was limited: age was missing for 30 participants (3.0%), gender for 6 (0.6%), relationship status for 2 (0.2%), parental status for 5 (0.5%), physician type (general practitioner vs specialist) for 17 (1.7%), career status (resident vs attending physician) for 17 (1.7%), and weekly working hours for 1 (0.1%). In addition, two participants who reported their gender as "Other" were not included in the women/men regression contrast. Overall, 59 participants had missing data on at least one required covariate; and two additional participants were excluded because of the gender

contrast, resulting in a complete-case analytic sample of 941 participants (93.9% of the study sample). All three final multivariable regression models therefore included N=941, as now reported in Table 4.

We also compared participants included in the regression analyses (n=941) with those not included (n=61). The groups did not differ in age, gender, relationship status, parental status, physician type, career status, or any of the three HDMC dimension scores (all p>0.05). Participants not included in the regression analyses reported slightly fewer weekly working hours than those included (46.55 vs 49.82 hours/week; Cohen's d=-0.27); the conventional equal-variance test yielded p=0.045, whereas the Welch test yielded p=0.063.

Given that only 6.1% of participants were excluded from the regression models, together with the comparison of included and non-included participants described above, we retained the complete-case approach rather than implementing multiple imputation. We have clarified this missing-data approach in the Methods and report the comparison of included and non-included participants in the Results.

The flow diagram currently provides no numerical breakdown for the 291 excluded respondents and refers vaguely to "the exact analytic sample." It should show explicit counts at every stage.

Response: Thank you for this comment. We have revised the participant flow diagram to provide explicit counts at each stage of participant inclusion. The revised figure now shows that 1292 physicians accessed the survey, 1238 provided informed consent, and 1002 had complete HDMC item data and constituted the study sample. It also reports the corresponding exclusions: 54 individuals did not provide consent, and 236 consenting participants did not have complete HDMC item data. Finally, the figure shows that 941 participants were included in the complete-case multivariable regression analyses; 59 participants were excluded because of missing data on at least one required covariate and two additional participants because their gender could not be included in the women/men regression contrast.

The manuscript should also report:

- **the recruitment dates;**
- **the exact numbers recruited from Belgium and France;**
- **whether country was included in, or tested as, a covariate;**
- **whether duplicate anonymous responses were prevented;**
- **a study-size or precision rationale; and**
- **the extent to which the achieved sample resembles the target physician population.**

Response: Thank you for these suggestions. We have expanded the reporting of recruitment, country of residence, sample size, and sample characteristics. Recruitment took place between 5 September and 5 November 2023. The country distribution is now reported in Table 1: 922 participants (92.0%) resided in Belgium, 65 (6.5%) in France, 2 (0.2%) in Switzerland, and 6 (0.6%) in other countries; country of residence was missing for 7 participants (0.7%).

Country was not included as a covariate in the primary models because the sample predominantly comprised participants residing in Belgium. We nevertheless conducted sensitivity analyses restricting the multivariable regression models to Belgian participants. Among the 922 Belgian participants, 868

had complete data for all regression variables, and the results were consistent with the primary analyses, with no substantive changes in the direction or overall pattern of associations.

Because responses were collected anonymously, no technical procedure was implemented to identify or prevent duplicate participation. We have now acknowledged this as a limitation of the online survey procedure.

The present analyses were secondary exploratory analyses of an existing dataset and were not specified when the original sample-size calculation was performed. Therefore, no separate a priori sample-size or power calculation was conducted for the present study. All 1002 participants with complete HDMC data were retained for the subgroup analyses, with 941 participants with complete covariate data included in the three primary multivariable regression models. Effect estimates and 95% confidence intervals are reported to convey the magnitude and precision of the observed associations.

Finally, we compared the achieved Belgian subsample descriptively with 2023 workforce data for active French-speaking physicians in Belgium provided by the Belgian National Institute for Health and Disability Insurance (INAMI/RIZIV; 2024). The distribution of general practitioners and specialists was closely aligned with the reference population (36.9% vs 35.7% general practitioners, respectively). However, our sample was younger (mean age 40.5 vs 51 years among general practitioners and 40.9 vs 49 years among specialists) and included a higher proportion of women (72.2% vs 49.9% among general practitioners and 62.3% vs 49.3% among specialists). We have therefore clarified in the revised manuscript that, although the sample was professionally diverse and closely resembled the reference population in its general practitioner/specialist distribution, it should not be considered representative of the Belgian physician population.

The phrase "a convenience sample representative of the broad physician population" should be revised. A convenience sample may be diverse, but representativeness cannot be assumed without a sampling frame and population comparison.

Response: Thank you for this important clarification. We agree that representativeness cannot be assumed from a convenience sample without a population-based sampling frame. We therefore replaced the phrase "a convenience sample representative of the broad physician population" with: "To recruit a broad and professionally diverse convenience sample, we used several approaches to encourage participation." As detailed in our response immediately above, comparison with Belgian workforce data showed some similarities but also important demographic differences; we therefore describe the sample as professionally diverse but not representative, and have strengthened the corresponding limitations regarding selection bias and generalisability.

Because recruitment included France as well as Belgium, a sensitivity analysis restricted to Belgian participants, or adjustment for country, would be useful. National differences in training, employment structures, and professional culture could influence HDMC scores.

Response: Thank you for this suggestion. As noted in our response above, we conducted the requested sensitivity analysis by re-estimating all three dimension-specific multivariable models among participants residing in Belgium. The analysis included 868 complete cases and yielded no substantive changes in the direction or overall pattern of associations. This sensitivity analysis is now reported in the Methods and Results.

<u>6. Multiplicity and the exploratory nature of the analyses need to be addressed</u>

A large number of comparisons were performed across four principal outcomes, numerous physician characteristics, seven subfactors, professional settings, and specialties. Bonferroni adjustment was applied to selected post-hoc comparisons, but there appears to be no correction or explicit multiplicity strategy for the broader set of tests. Please clearly identify which outcomes and comparisons were primary, secondary, or exploratory. Either an appropriate multiplicity approach, such as false-discovery-rate control, should be considered, or the analyses should be consistently presented as exploratory, with emphasis on estimates, confidence intervals, and effect sizes rather than binary statistical significance.

Response: Thank you for this important comment. We agree that the large number of comparisons requires a clearer distinction between the principal and complementary exploratory analyses. We have revised the Methods to clarify that comparisons involving the three HDMC dimensions across the main physician characteristics constitute the principal subgroup analyses reported in Table 3. Given the absence of prior empirical evidence regarding subgroup differences in HDMC endorsement, these analyses are presented as exploratory and hypothesis-generating rather than confirmatory, with no prespecified directional hypotheses.

Effect sizes (Cohen's *d* for two-group comparisons and eta-squared [η^2] for comparisons involving more than two groups) have been added to Table 3 to facilitate interpretation of the magnitude of observed differences. For post-hoc pairwise comparisons following the principal ANOVAs, Bonferroni correction was applied following standard ANOVA and Games-Howell comparisons were used following Welch's ANOVA where appropriate.

Analyses involving the five first-order HDMC subfactors retained in the revised 20-item model (online supplemental table 3) and additional physician characteristics, including working context (online supplemental tables 4a, 4b, 5a and 5b), are explicitly identified as complementary exploratory analyses. Given their exploratory nature and the large number of comparisons, no post-hoc pairwise comparisons were conducted for the first-order subfactor analyses. Analyses by medical specialty remain descriptive because of the small and unequal group sizes (online supplemental table 6).

We did not apply a global multiplicity correction across all subgroup tests. Instead, we now explicitly caution against interpreting individual p-values as confirmatory evidence and emphasise effect sizes and the overall pattern of findings rather than statistical significance alone.

The OSF registration is described as registration of the broader research project. Please state explicitly whether the research question, subgroup analyses, variable dichotomisations, and regression models in the present manuscript were preregistered before examining the data. If not, they should be labelled as post-hoc exploratory analyses.

Response: Thank you for this important comment. We have clarified the distinction between the broader preregistered research project and the analyses reported in the present manuscript. The OSF preregistration specified the development and examination of the structure of medical-culture dimensions and their associations with physicians' mental health and health behaviours. It also anticipated exploratory analyses examining whether associations between medical culture and these outcomes varied according to sociodemographic and professional characteristics, including gender, age, physician type, career status, seniority, and country of residence. However, the specific research question addressed in the present manuscript (whether endorsement of the HDMC dimensions differs across physician subgroups) was not preregistered. The subgroup comparisons, variable categorisations, and multivariable regression models reported in the present study were also not

prespecified in the OSF registration. We have revised the manuscript to state this explicitly and to consistently describe the analyses as post-hoc exploratory and hypothesis-generating rather than confirmatory.

As this is at least the third publication using the same dataset, the authors should provide a concise statement specifying the distinct aims, variables, and analyses of each publication and explaining how duplicate reporting has been avoided.

Response: Thank you for this comment. As noted in response to the Editor, we added a concise statement in the Methods distinguishing the three publications based on this dataset. The first focused on HDMC development/psychometric evaluation and burnout; the second on HDMC associations with mental health outcomes and health-related behaviours; and the present study on variation in HDMC endorsement according to physicians' demographic and professional characteristics using subgroup comparisons and multivariable analyses. The primary research questions, variables of interest, and principal analyses are therefore distinct, and the present analyses do not duplicate those of the previous publications.

7. Effect sizes and practical importance should receive more attention

With approximately 1000 participants, relatively small differences can achieve very low p values. The manuscript should report effect sizes for the unadjusted analyses, such as Cohen's d and η^2 or ω^2, and preferably standardised coefficients or partial R^2 for regression terms. For example, a global-score difference of approximately 0.15–0.20 points on a 1–5 scale may be statistically robust but modest in practical magnitude. The Discussion should distinguish statistical significance from meaningful cultural or clinical differences. The relatively low adjusted R^2 values of 0.04–0.12 also indicate that most variation in HDMC endorsement remains unexplained.

Response: Thank you for this suggestion. We have added effect-size estimates to both the unadjusted and adjusted analyses. For the unadjusted subgroup comparisons, Cohen's *d* is now reported for two-group comparisons and eta-squared (η^2) for comparisons involving more than two groups (Table 3). For the multivariable regression analyses, we have added standardized regression coefficients (β) alongside the unstandardized coefficients (*B*), 95% confidence intervals, and *p*-values in Table 4. This allows the magnitude of the observed differences and adjusted associations to be considered alongside their statistical significance. As detailed in response to Comment 3, the global HDMC composite is no longer included in the inferential subgroup comparisons or regression analyses.

We have also revised the Discussion to place greater emphasis on the magnitude and practical interpretation of the findings. In particular, we acknowledge that the observed subgroup differences were generally small to moderate and should not be interpreted as evidence of substantial cultural differences between physician groups solely on the basis of statistical significance. We also note that the modest proportion of variance explained by the multivariable models indicates that most variation in HDMC endorsement remains unexplained by the demographic and professional characteristics examined in this study.

8. Several conclusions exceed what can be inferred from this cross-sectional analysis

Terms including "antecedents," "risk factors," "predictors," "vulnerable," and "contribute to" imply temporal or causal relationships that are not established here. These should generally be replaced by "associated factors," "correlates," or "groups reporting higher scores."

In particular:

- **Long working hours may reinforce self-sacrificing norms, but physicians who endorse such norms may also choose or accept longer hours.**
- **Cross-sectional age differences cannot distinguish ageing effects, cohort effects, career selection, or survivor effects.**
- **The general practitioner–specialist contrast may reflect work setting, employment arrangements, workload, or organisational culture rather than specialty-specific socialisation.**
- **The finding that younger physicians score higher on some dimensions does not establish that they are more likely to "internalise" norms during training.**
- **"Strong endorsement" should not be used unless validated thresholds or normative categories exist.**

Response: Thank you for this important comment. We agree that the cross-sectional design does not support temporal or causal interpretations. We have revised the terminology throughout the manuscript accordingly, replacing terms such as "predictors," "risk factors," "antecedents," "vulnerable," and "contribute to," where they referred to findings from the present study, with non-causal formulations such as "associated with," "physician characteristics," and "groups reporting higher scores." The multivariable analyses are likewise described as examining associations while adjusting for the other characteristics included in the models, rather than as identifying predictors.

We have also revised the Discussion to reflect the alternative explanations inherent in the cross-sectional design. Specifically, we now acknowledge the potentially bidirectional relationship between weekly working hours and endorsement of professional cultural norms; clarify that the observed age differences cannot distinguish ageing effects from cohort effects, career selection, or survivor effects; and acknowledge that differences between general practitioners and specialists may reflect work setting, employment arrangements, workload, or organisational culture rather than specialty-specific socialisation. We no longer interpret the higher scores observed among younger physicians on some HDMC dimensions as evidence that they are more likely to internalise these norms during training. Finally, we have removed references to "strong endorsement," as no validated thresholds or normative categories are available for interpreting HDMC scores.

The conclusions also group younger physicians, female physicians, specialists, and physicians with long working hours together as endorsing norms of self-sacrifice, invulnerability, and burnout stigma. The actual associations are dimension-specific. For example, gender is principally associated with Professional Commitment and the Myth of the Invulnerable Physician, whereas age is associated with invulnerability and stigma. The abstract and Discussion should map each characteristic to the relevant outcome rather than implying that all groups scored higher on all dimensions.

Response: We agree that the observed associations are dimension-specific and should not be summarised as though each physician characteristic were associated with all three HDMC dimensions. This concern also informed our decision, described in response to Comment 3, to remove the global HDMC composite from the inferential analyses and to focus the subgroup comparisons and multivariable models on the three distinct HDMC dimensions. We have revised the Abstract, Results, Discussion, and Conclusion accordingly so that each physician characteristic is mapped to the specific HDMC dimension(s) with which it was associated, rather than implying a common pattern across all dimensions or summarising the findings through an overall HDMC score.

9. The conceptual meaning of “Physicians’ Professional Commitment” needs greater nuance

Professional commitment can be adaptive and central to good patient care. The measured dimension appears to combine work–life interference, sacrifice, and centrality of professional identity. The authors should clarify why a high score is interpreted as harmful and distinguish healthy professional commitment from maladaptive overcommitment or self-sacrificing commitment.

Otherwise, describing physicians with high professional commitment as endorsing a “harmful dimension” may inadvertently pathologise professional dedication. A more precise dimension label may be warranted.

Response: Thank you for this important comment. We agree that professional commitment is not inherently harmful and that the terminology could otherwise be interpreted as pathologizing dedication to medicine. In the original psychometric study, “Physicians’ Professional Commitment” was the label assigned to a second-order dimension comprising three empirically identified first-order factors: Work Priority Strain, the Self-Sacrificing Nature of Medical Practice, and the Physician’s Central Identity Role. The construct measured by this dimension therefore does not refer to professional commitment in its broad or inherently adaptive sense, but to a specific pattern characterised by work-life interference, perceived personal sacrifice, and the centrality of medicine to personal identity.

Importantly, higher scores on this HDMC dimension have previously been associated not only with burnout but also with depressive symptoms and several potentially problematic health-related practices, including reduced help-seeking, self-diagnosis/self-treatment, and psychoactive medication use. Although these associations were generally modest and do not imply causality, they suggest that the specific form of professional commitment captured by the HDMC is not uniformly adaptive.

Because the dimension label and factor structure were established in the original psychometric study, we retained “Physicians’ Professional Commitment” for consistency with the existing measure. We have, however, revised the manuscript to explicitly acknowledge that professional commitment can be a source of meaning, fulfilment, and high-quality patient care, and to clarify that higher HDMC scores reflect the specific work-prioritising, self-sacrificing, and identity-central aspects captured by this dimension rather than professional commitment per se.

Minor comments

The Results heading “Hierarchical regressions” is inconsistent with the Methods, which describe simultaneous entry using the enter method. These are multivariable linear regressions, not hierarchical regressions.

Response: Thank you for identifying this inconsistency. All physician characteristics were entered simultaneously in a single block using the enter method; the analyses are therefore multivariable linear regressions rather than hierarchical regressions. We have changed the Results heading accordingly and have used consistent terminology throughout the Methods, Results, and Table 4.

The Table 3 legend states that “correlation is significant.” These are group-comparison tests, not correlations.

Response: Thank you for noting this error. These analyses are group-comparison tests rather than correlations. We have removed the wording “correlation is significant” from the Table 3 legend. The

revised table reports the test statistics, effect sizes, and corresponding two-sided *p*-values directly, without significance asterisks.

Please specify which analyses used Welch's ANOVA. If homogeneity of variance was violated, clarify which post-hoc procedure was used; standard equal-variance pairwise procedures may not be appropriate, and Games–Howell comparisons may be preferable.

Response: Thank you for this suggestion. We have clarified the decision rule in the Statistical analyses section. Homogeneity of variance was assessed using Levene's test. Standard one-way ANOVA was used when the assumption was met; when Levene's test indicated unequal variances, Welch's ANOVA was reported. Post-hoc comparisons for the principal subgroup analyses in Table 3 used Bonferroni adjustment following standard ANOVA and Games-Howell comparisons following Welch's ANOVA. No post-hoc pairwise comparisons were conducted for the exploratory subfactor analyses reported in online supplemental table 3.

The supplementary mean plots should include uncertainty intervals. Point-and-error-bar plots would be more informative than connected lines, which may imply continuous trajectories across arbitrary categories.

Response: Thank you for this suggestion. We have revised online supplemental figures 1 and 2 accordingly. The previous connected mean plots have been replaced with point-and-error-bar plots showing mean HDMC scores and 95% confidence intervals for each age and weekly working hours category. Connecting lines were removed to avoid implying continuous trajectories across categorical groups.

"Most physicians worked over 40 hours" should be "40 hours or more" if the category includes exactly 40 hours.

Response: Thank you for pointing this out. The reviewer is correct that the category includes physicians working exactly 40 hours per week. We have therefore changed "Most physicians worked over 40 hours" to "Most physicians worked 40 hours or more" in the Results section.

Please use "gender" and "sex" consistently and explain exactly how the variable was assessed. The Discussion currently refers to "female sex," whereas the variable is labelled gender.

Response: We agree and have revised the terminology throughout the manuscript. The questionnaire assessed self-reported gender, with the response options "woman", "man", and "other"; biological sex was not assessed. We therefore consistently use the term gender rather than sex throughout the revised manuscript. Analyses comparing women and men excluded participants selecting "other" because this group was too small for meaningful subgroup analysis.

"Trial registration" is not the most appropriate label for an observational survey. "Study preregistration" would be clearer.

Response: Thank you for this suggestion. We agree that "Study preregistration" is more appropriate for this observational survey and have replaced "Trial registration" with "Study preregistration" in the Abstract.

The keyword "risk factors" should be replaced with "associated factors" or "physician characteristics."

Response: Thank you for this suggestion. We agree that "risk factors" may imply a causal relationship and have replaced this keyword with **"**physician characteristics."

The abstract's description of "primary and secondary outcome measures" does not distinguish primary from secondary outcomes.

Response**:** Thank you for this comment. Because the analyses reported in the present study were exploratory and the three HDMC dimension scores were not prespecified as primary versus secondary outcomes, we have revised this section of the Abstract to describe the outcomes assessed without implying a prespecified primary/secondary hierarchy.

In the Acknowledgements, "participated to the online survey" should be "participated in the online survey." Because two individuals are acknowledged, "for her help" should be "for their help."

Response: We have corrected both wording issues in the Acknowledgements ("participated in the online survey" and "for their help").

Specialty terminology should be reviewed, for example, "obstetrics and gynaecology" rather than "gynaecology and obstetrical."

Response: Thank you for noting this terminology issue. We have corrected "gynaecology and obstetrical" to "obstetrics and gynaecology" in Table 1.

The STROBE checklist appears to state that interactions and a study-size explanation are reported, although these are not evident in the manuscript. The checklist should be corrected.

Response: Thank you for identifying these inconsistencies. We have revised the STROBE checklist to ensure that it accurately reflects the revised manuscript. We corrected the entry concerning interaction analyses, as no interaction analyses were performed in the present study. We also added a study-size explanation to the Methods, clarifying that the present analyses were secondary exploratory analyses of an existing dataset and that no separate a priori sample-size calculation was conducted for the specific analyses reported here. The corresponding STROBE checklist entry has been updated accordingly.

<u>Reviewer: 2</u>

Dr. Mihriye Mete, MedStar Health Research Institute, Georgetown University Medical Center

Comments to the Author: The empirical exploration of the culture in which physicians operate is a significant contribution to the literature. The paper is very well written. Statistical analyses are appropriate.

Please clarify the following:

- **Hierarchical regressions are mentioned in the results but the stats plan section does not explain this approach. The analyses can be redone with using linear mixed methods with random effects at the organizational level. Given the snowball nature of the survey design/data collection, it becomes even more important to control for unexplained factors at the organizational level that are strongly correlated with culture--potentially.**

Response: Thank you for this important comment. As also noted in response to Reviewer 1, the term "hierarchical regressions" in the original submission was incorrect. All physician characteristics were entered simultaneously using the enter method; the analyses are therefore multivariable linear

regressions. We corrected this terminology throughout and clarified the analytical approach in the Statistical analyses section.

We agree that physicians working within the same organisation may share organisational characteristics that could influence their endorsement of medical cultural norms and that accounting for such clustering would be informative. However, participants' organisations were intentionally not collected in this anonymous survey to minimise potentially identifying information. We are therefore unable to identify physicians belonging to the same organisation or to estimate organisation-level random effects. Although the present study examines medical culture at the level of the profession rather than organisational culture, we recognise that professional cultural norms may vary across and interact with organisational contexts. We have therefore explicitly acknowledged the inability to account for potential clustering and variation across organisations as a limitation of the study.

While the new scale is theoretically meaningful, overall results (including in the primary publication showing correlations with burnout) suggest that it is not strongly related to burnout (weak correlations with burnout) and that the effect sizes for the different categories of the physicians in relation to the subscales are practically negligible. These results lead to questions about the validity of the scale, especially construct validity.

Response: Thank you for raising this important point. We agree that the generally small to moderate effect sizes observed in the present subgroup analyses should be interpreted cautiously and do not support strong claims regarding substantial differences in HDMC endorsement between physician groups. We have revised the manuscript accordingly and now place greater emphasis on effect sizes and their magnitude rather than on statistical significance alone.

We would, however, distinguish the magnitude of associations with burnout from the construct validity of the HDMC measure. The HDMC was developed to assess physicians' endorsement of potentially harmful professional cultural norms rather than burnout itself. Associations with burnout therefore provide one source of evidence regarding the relationship between the HDMC and a theoretically relevant external construct, but do not constitute a comprehensive test of construct validity. In the original psychometric study, the structure of the HDMC was examined using exploratory and confirmatory factor analyses, alongside its associations with burnout.

We nevertheless agree that the existing psychometric evidence remains preliminary. As detailed in response to Reviewer 1, the measurement-invariance analyses conducted during this revision led to a post-hoc refinement of the model, resulting in the revised 20-item specification used here. We have therefore strengthened the Discussion to state that this revised model requires independent psychometric evaluation, including its factorial structure, reliability, measurement invariance, and convergent, discriminant, and criterion-related validity, and we avoid describing the HDMC as comprehensively validated.

Given the nature of the survey design, selection bias is a serious problem that endangers the validity of the inference. This can't be addressed here but the authors might want to consider reaching out to other physician wellbeing researchers to further validate their scale in larger and more systematically collected samples (Stanford group for instance).

Response: We agree that the convenience and partly snowball-based recruitment strategy may have introduced selection bias, particularly if physicians with greater interest in medical culture or physician well-being were more likely to participate. This limitation cannot be resolved retrospectively and has

been made more explicit in the revised Discussion. As detailed in response to Reviewer 1, comparison with available Belgian workforce data showed both similarities and demographic differences; we therefore explicitly state that the sample should not be considered representative of the target physician population.

We also agree that independent replication is important, particularly following the post-hoc refinement of the measurement model. The Discussion now emphasises the need to evaluate the revised 20-item model in independent, larger, and more systematically recruited physician samples.

VERSION 2 - REVIEW

**Reviewer**	**1**
**Name**	**Son, Daisuke**
**Affiliation**	**Tottori University, Department of Community-based Family Medicine, Faculty of Medicine**
**Date**	**13-Sep-2026**
**COI**	

Thank you for the careful and extensive revision. The manuscript has improved substantially, and the authors have addressed most of my previous concerns in a thoughtful and transparent manner. In particular, I appreciate the comprehensive re-audit of the analyses, the use of continuous age and working-hours variables in the primary regression models, the addition of effect sizes and standardised coefficients, the clearer reporting of missing data and analytic sample size, and the more cautious interpretation of cross-sectional findings. The decision to restrict inferential analyses to the three HDMC dimensions and retain the global composite for descriptive purposes only is also appropriate.

The additional measurement-invariance analyses are an important improvement. The authors are appropriately transparent that full scalar invariance was not achieved and that the revised 20-item model resulted from a post-hoc refinement during peer review. I have only a small number of remaining issues, primarily concerning numerical consistency and reporting.

1. Please conduct one final reconciliation of participant counts and denominators

There still appear to be some inconsistencies between the response letter and the revised tables.

The response letter states that the physician-type variable identified 645 specialists, of whom specialty information was available for 627. However, Table 3 reports 646 specialists and 339 general practitioners. Similarly, Table 1 contains 457 attending specialists plus 278 attending general practitioners (=735 attending physicians), whereas Table 3 reports 736 attending physicians.

These discrepancies may reflect the way the binary “physician type” and “career status” variables were derived from the original multicategory variable, but if so, this recoding should be explicitly explained. Otherwise, the counts should be corrected.

There is also an apparent denominator inconsistency within the specialty section of Table 1. The table is labelled N=645, and most percentages appear to use 645 as the denominator (e.g., 170/645=26.4% for internal medicine), whereas 25 physicians in obstetrics and gynaecology are reported as 4.0%, which corresponds approximately to the 627 participants with available specialty data rather than 645. Please use one clearly stated denominator consistently, or explicitly indicate that percentages exclude missing specialty data.

Given the numerical inconsistencies identified in the original submission, I would strongly encourage one final automated cross-check of all Ns, percentages, and table-to-text values.

2. Measurement invariance: clarify the implications for observed-score comparisons

The new analyses show configural and metric invariance across all four comparisons, but full scalar invariance was not supported. Partial scalar invariance required freeing one intercept for gender, five for age, three for resident/attending status, and three for GP/specialist status.

The manuscript appropriately acknowledges this limitation. However, because the principal subgroup analyses continue to compare observed composite scores, it would be helpful to clarify that partial scalar invariance does not necessarily guarantee that all observed-score differences are free from measurement-related influences, particularly for the age comparison where five intercepts were freed.

If feasible, a sensitivity analysis using latent mean comparisons or scores excluding the non-invariant items would further strengthen the conclusions. I would not regard this as essential for publication given the explicitly exploratory nature of the study, but the limitation of observed-score comparisons under partial rather than full scalar invariance should be stated as clearly as possible.

Please also specify the estimator used for the continuous-indicator SEM and briefly describe how the particular intercepts to be freed were selected during the iterative partial-invariance procedure.

3. Please standardise the direction of Cohen’s d

The signs of Cohen’s d are not always presented consistently between Table 3 and the narrative. For example, the table compares men with women and reports d=-0.56, whereas the text states that women scored higher while retaining d=-0.56. For parental status and career status, by contrast, the narrative appears to reverse the sign when reversing the direction of comparison.

This does not affect the substantive results, but the convention should be made consistent. One simple approach would be to state explicitly in the Table 3 footnote that d is calculated

as the first listed group minus the second listed group and retain that convention throughout, or alternatively report the absolute magnitude of d in the narrative while describing the direction verbally.

4. Please report greater numerical precision for continuous predictors in Table 4

For continuous age and weekly working hours, rounding the unstandardised coefficients and confidence intervals to two decimal places makes some estimates difficult to interpret. For example, Table 4 reports values such as 0.00 (-0.00 to 0.01) and 0.01 (0.00 to 0.01), whereas the supplemental table shows the underlying estimates more informatively as 0.003, −0.019, 0.005, etc.

I suggest reporting B and its 95% CI to three decimal places for age and weekly working hours.

5. A few final reporting details

In the response letter, the authors state that gender was self-reported using the categories “woman”, “man”, and “other”. However, I could not identify this explicit description in the Methods of the revised manuscript; the manuscript currently states only that gender was collected. Please add the response options and clarify that biological sex was not assessed, consistent with the response letter.

Finally, please carefully clean the accepted-changes version before publication. The marked manuscript contains several apparent remnants of earlier citation formats or tracked edits—for example, parenthetical author-year citations appearing alongside numbered citations in the Discussion. These appear editorial rather than substantive but should be removed from the clean version.

Overall assessment:

The authors have satisfactorily addressed the substantive methodological concerns raised in my initial review. The remaining issues are primarily matters of numerical reconciliation, methodological clarification, and reporting. Subject to these relatively minor revisions, I would support publication.

VERSION 2 - AUTHOR RESPONSE

Reviewer: 1 - Dr. Daisuke Son, Tottori University

Comments to the Author:

Thank you for the careful and extensive revision. The manuscript has improved substantially, and the authors have addressed most of my previous concerns in a thoughtful and transparent manner. In particular, I appreciate the comprehensive re-audit of the analyses, the use of continuous age and working-hours variables in the primary regression models, the addition of effect sizes and standardised coefficients, the clearer reporting of missing data and analytic sample size, and the more cautious interpretation of cross-sectional findings. The decision to

restrict inferential analyses to the three HDMC dimensions and retain the global composite for descriptive purposes only is also appropriate.

The additional measurement-invariance analyses are an important improvement. The authors are appropriately transparent that full scalar invariance was not achieved and that the revised 20-item model resulted from a post-hoc refinement during peer review. I have only a small number of remaining issues, primarily concerning numerical consistency and reporting.

Response : We thank the reviewer for their careful reassessment of the revised manuscript and for these additional suggestions. We have addressed each point below and updated the manuscript, tables, and supplemental material accordingly.

1. Please conduct one final reconciliation of participant counts and denominators

There still appear to be some inconsistencies between the response letter and the revised tables. The response letter states that the physician-type variable identified 645 specialists, of whom specialty information was available for 627. However, Table 3 reports 646 specialists and 339 general practitioners. Similarly, Table 1 contains 457 attending specialists plus 278 attending general practitioners (=735 attending physicians), whereas Table 3 reports 736 attending physicians. These discrepancies may reflect the way the binary "physician type" and "career status" variables were derived from the original multicategory variable, but if so, this recoding should be explicitly explained. Otherwise, the counts should be corrected.

There is also an apparent denominator inconsistency within the specialty section of Table 1. The table is labelled N=645, and most percentages appear to use 645 as the denominator (e.g., 170/645=26.4% for internal medicine), whereas 25 physicians in obstetrics and gynaecology are reported as 4.0%, which corresponds approximately to the 627 participants with available specialty data rather than 645. Please use one clearly stated denominator consistently, or explicitly indicate that percentages exclude missing specialty data. Given the numerical inconsistencies identified in the original submission, I would strongly encourage one final automated cross-check of all Ns, percentages, and table-to-text values.

Response : Thank you for identifying these discrepancies. We re-examined the participant-level survey response and the variables used in Tables 1 and 3.

Table 1 classifies 15 participants in the combined "occupational medicine/insurance physician/ school physician/medical consultant" category. One of these participants had selected "specialist physician" in the survey and specified "occupational medicine" as their specialty, although occupational medicine was also available as a separate physician-type response option. We classified this participant under the occupational medicine category for the descriptive breakdown in Table 1, while retaining their explicitly selected specialist status for the binary GP-versus-specialist comparison in Table 3. This accounts for the 645 physicians in the two specialist categories of Table 1 versus 646 specialists in Table 3. The 339 general practitioners are the same in both classifications. Of the 645 physicians in the specialist categories of Table 1, 627 provided specialty information and 18 did not. We have clarified these different classifications in the manuscript and table notes.

We also found that the same participant had been assigned to the attending group on the basis of age, although attending versus resident status had not been directly reported. We have removed this inferred classification and repeated the attending-versus-resident comparisons reported in

Table 3. The corrected groups comprise 735 attending physicians and 249 residents, consistent with the categories shown in Table 1. We have updated the corresponding Table 3 results and Results text. These corrections did not change the direction or statistical significance of any of the attending-versus-resident comparisons in Table 3, and our interpretation of these comparisons remains unchanged. We also re-estimated the multivariable regression models, the age and working-hours sensitivity models, the Belgium-only analyses, and the career-status measurement-invariance models using the corrected career status variable. The corresponding results have been updated in Table 4 and online supplemental tables 7 and 8.

For the specialty breakdown in Table 1, we have specified that percentages use all 645 physicians in the two specialist categories as the denominator, including the 18 with missing specialty information. We corrected the obstetrics and gynaecology entry from 25 (4.0%) to 25 (3.9%).

We then cross-checked the revised participant counts and denominators across Tables 1, 3 and 4, the relevant supplemental tables, and the corresponding manuscript text. We updated the affected values consistently following the correction of this participant's career status.

2. Measurement invariance: clarify the implications for observed-score comparisons

The new analyses show configural and metric invariance across all four comparisons, but full scalar invariance was not supported. Partial scalar invariance required freeing one intercept for gender, five for age, three for resident/attending status, and three for GP/specialist status. The manuscript appropriately acknowledges this limitation. However, because the principal subgroup analyses continue to compare observed composite scores, it would be helpful to clarify that partial scalar invariance does not necessarily guarantee that all observed-score differences are free from measurement-related influences, particularly for the age comparison where five intercepts were freed. If feasible, a sensitivity analysis using latent mean comparisons or scores excluding the non-invariant items would further strengthen the conclusions. I would not regard this as essential for publication given the explicitly exploratory nature of the study, but the limitation of observed-score comparisons under partial rather than full scalar invariance should be stated as clearly as possible. Please also specify the estimator used for the continuous-indicator SEM and briefly describe how the particular intercepts to be freed were selected during the iterative partial-invariance procedure.

Response: Thank you for this helpful comment. We have revised the Methods to specify that the continuous-indicator multigroup SEM models were estimated using maximum likelihood. We have also clarified how partial scalar invariance was evaluated: after a full scalar model failed to meet the prespecified fit criteria, we examined Stata's score tests for group invariance, freed the item intercept with the largest score-test statistic, and re-estimated the model. We repeated this procedure until the criteria for partial scalar invariance were met. We have expanded the Discussion to clarify that partial scalar invariance does not establish that differences in observed composite scores are entirely free from measurement-related influences. This concern is particularly relevant to the age comparison, for which five item intercepts were freed. We therefore interpret the subgroup comparisons as exploratory. We have not added latent-mean or item-exclusion sensitivity analyses, which the reviewer indicated were not essential for publication.

3. Please standardise the direction of Cohen's d

The signs of Cohen's d are not always presented consistently between Table 3 and the narrative. For example, the table compares men with women and reports d=-0.56, whereas the text states that women scored higher while retaining d=-0.56. For parental status and career status, by contrast, the narrative appears to reverse the sign when reversing the direction of comparison. This does not affect the substantive results, but the convention should be made consistent. One simple approach would be to state explicitly in the Table 3 footnote that d is calculated as the first listed group minus the second listed group and retain that convention throughout, or alternatively report the absolute magnitude of d in the narrative while describing the direction verbally.

Response: Thank you for pointing out this inconsistency. We have standardised the reporting of Cohen's *d*. In Table 3, signed values follow a single convention: the mean of the first group listed minus that of the second group listed. We have stated this convention in the table note and corrected the sign of the effect size for the relationship-status comparison on Stigma towards Burnout. In the Results narrative, we now describe the direction of each difference in words and report the absolute magnitude as $|d|$, without reversing or carrying over the table's sign when the groups are discussed in a different order. We checked all two-group comparisons in Table 3 and their corresponding descriptions for consistency.

4. Please report greater numerical precision for continuous predictors in Table 4

For continuous age and weekly working hours, rounding the unstandardised coefficients and confidence intervals to two decimal places makes some estimates difficult to interpret. For example, Table 4 reports values such as 0.00 (-0.00 to 0.01) and 0.01 (0.00 to 0.01), whereas the supplemental table shows the underlying estimates more informatively as 0.003, −0.019, 0.005, etc. I suggest reporting B and its 95% CI to three decimal places for age and weekly working hours.

Response: Thank you for this suggestion. We have revised Table 4 to report the unstandardised coefficients (B) and their 95% confidence intervals to three decimal places for age and weekly working hours in all three regression models. This makes the direction and magnitude of estimates clearer where rounding to two decimal places previously produced values such as 0.00 or −0.00. As explained in our response to Comment 1, one participant was no longer eligible for the complete-case regression analyses because their career status was treated as missing. We re-estimated all three models (n=940 instead of n=941). This accounts for the small changes in some other Table 4 estimates; the substantive interpretation of the regression findings did not change.

5. A few final reporting details

In the response letter, the authors state that gender was self-reported using the categories "woman", "man", and "other". However, I could not identify this explicit description in the Methods of the revised manuscript; the manuscript currently states only that gender was collected. Please add the response options and clarify that biological sex was not assessed, consistent with the response letter.

Response : Thank you for pointing out this omission. We have added the response options to the Methods section and clarified that gender was self-reported as "woman", "man", or "other", while biological sex was not assessed.

Finally, please carefully clean the accepted-changes version before publication. The marked manuscript contains several apparent remnants of earlier citation formats or tracked edits—for example, parenthetical author-year citations appearing alongside numbered citations in the Discussion. These appear editorial rather than substantive but should be removed from the clean version.

Response : Thank you for pointing this out. We have reviewed the clean manuscript to remove remnants of tracked changes and earlier citation formats, including parenthetical author–year citations appearing alongside numbered references in the Discussion. We have also checked the manuscript for consistent reference formatting.

Overall assessment: The authors have satisfactorily addressed the substantive methodological concerns raised in my initial review. The remaining issues are primarily matters of numerical reconciliation, methodological clarification, and reporting. Subject to these relatively minor revisions, I would support publication.
