# Supplementary material for: Harmful dimensions of medical culture across physician subgroups: a cross-sectional study of 1002 physicians predominantly practising in Belgium
Source: BMJ Open. 2026 Oct 20;16(10):e124359. doi: 10.1136/bmjopen-2026-124359 (PMC13684193; doi:10.1136/bmjopen-2026-124359)
Supplement: Supplementary file 2 [file bmjopen-16-10-s001.docx]

**Online supplemental table 1.** Descriptive statistics for the revised 20-item HDMC dimensions, retained sub-dimensions, and global score.

| **HDMC** | **Related sub-dimensions** | **# of items** | **Mean** | **SD** | **α** |
| --- | --- | --- | --- | --- | --- |
| **Physicians’ Professional Commitment** | | 11 | 3.68 | 0.64 | 0.85 |
|  | Work Priority Strain | 4 | 3.90 | 0.82 | 0.87 |
|  | Physician’s Central Identity Role | 4 | 3.04 | 0.84 | 0.78 |
|  | Sacrificial Nature of Medical Practice | 3 | 4.23 | 0.78 | 0.86 |
| **Myth of the Invulnerable Physician** | | 6 | 3.64 | 0.75 | 0.77 |
|  | Physician’s Moral Obligation to Patients and Colleagues | 4 | 3.63 | 0.85 | 0.78 |
|  | Physician’s Discomfort with Patient Role | 2 | 3.67 | 1.00 | 0.72 |
| **Stigma towards Burnout** | | 3 | 2.51 | 0.85 | 0.61 |
| **HDMC global score** | | 20 | 3.49 | 0.54 | 0.85 |

**Note**. N=1002. SD, standard deviation; α, Cronbach’s alpha. The revised 20-item HDMC model excludes item ec_10 from the Stigma towards Burnout dimension. Consequently, this dimension comprises three items and is represented as a single dimension without the two sub-dimensions specified in the original 21-item model. The sub-dimensions reported for Physicians’ Professional Commitment and Myth of the Invulnerable Physician are unchanged from the original model.

**Online supplemental figure 1**. Mean scores and 95% confidence intervals from the revised 20-item model for the three HDMC dimensions according to age categories.

**Note.** Points represent mean dimension scores; error bars represent 95% confidence intervals. Scores are based on the revised 20-item HDMC model.

**Online supplemental figure 2**. Mean scores and 95% confidence intervals for the three HDMC dimensions from the revised 20-item model according to weekly working hours categories.

**Note**. Points represent mean dimension scores; error bars represent 95% confidence intervals. Scores are based on the revised 20-item HDMC model**.**

**Online supplemental table 2.** Post-hoc pairwise comparisons for HDMC dimension scores from the revised 20-item model by age and weekly working-hours categories

| **HDMC** | **Group variable** | **Group comparison** | **Mean difference** | **95% CI** | **p-value** |
| --- | --- | --- | --- | --- | --- |
| **Physician’s Professional Commitment** | | | | | |
|  | **Weekly working hours** | | | | |
|  |  | <30 vs > 50 | -0.58 | (-0.90 to -0.26) | <0.001 |
|  |  | 30-39 vs 40-50 | -0.24 | (-0.39 to -0.08) | <0.001 |
|  |  | 30-39 vs >50 | -0.56 | (-0.71 to -0.41) | <0.001 |
|  |  | 40-50 vs >50 | -0.32 | (-0.43 to -0.22) | <0.001 |
| **Myth of the Invulnerable Physician** | | | | | |
|  | **Age category** | | | | |
|  |  | <35 vs ≥ 65 | 0.89 | (0.56 to 1.21) | <0.001 |
|  |  | <35 vs 45-54 | 0.27 | (0.10 to 0.44) | <0.001 |
|  |  | <35 vs 55-64 | 0.62 | (0.40 to 0.84) | <0.001 |
|  |  | 35-44 vs ≥65 | 0.73 | (0.39 to 1.07) | <0.001 |
|  |  | 35-44 vs 55-64 | 0.46 | (0.22 to 0.71) | <0.001 |
|  |  | 45-54 vs 55-64 | 0.35 | (0.10 to 0.60) | <0.001 |
|  |  | 45-54 vs ≥65 | 0.62 | (0.27 to 0.96) | <0.001 |
| **Stigma Towards Burnout** | | | | | |
|  | **Age category** | | | | |
|  |  | <35 vs 35-44 | 0.21 | (0.01 to 0.42) | 0.030 |
|  |  | <35 vs 45-54 | 0.34 | (0.14 to 0.54) | <0.001 |
|  |  | <35 vs 55-64 | 0.42 | (0.20 to 0.64) | <0.001 |
|  |  | <35 vs ≥65 | 0.40 | (0.04 to 0.77) | 0.024 |
|  | **Weekly working hours** | | | | |
|  |  | 30-39 vs >50 | -0.28 | (-0.51 to -0.05) | 0.008 |
|  |  | 40-50 vs >50 | -0.21 | (-0.36 to -0.06) | 0.002 |

**Note**. Only statistically significant post-hoc pairwise comparisons are reported. Mean differences are expressed as the mean of the first group minus the mean of the second group. Bonferroni-adjusted pairwise comparisons were used following standard ANOVA when the homogeneity of variance assumption was met; Games–Howell comparisons were used following Welch’s ANOVA when this assumption was violated. CI, confidence interval; HDMC, Harmful Dimensions of Medical Culture.

**Online supplemental table 3**. Exploratory subgroup comparisons of HDMC sub-dimension scores.

|  | **N** | **Work priority strain  (Mean, SD)** | **t (p) or F (p)** | **Central Identity Role**  **(Mean, SD)** | **t (p) or F (p)** | **Sacrificial Nature of Medical Practice** **(Mean, SD)** | **t (p) or F (p)** | **Moral Obligation - Patients and Colleagues (Mean, SD)** | **t (p) or F (p)** | **Discomfort with Patient Role (Mean, SD)** | **t (p) or F (p)** |
| --- | --- | --- | --- | --- | --- | --- | --- | --- | --- | --- | --- |
| **Gender** | | | | | | | |  | | | |
| Man | 347 | 3.87 ± 0.83 | t = - 0.93,  p = 0.352 | 3.03 ± 0.81 | t = -0.27, p = 0.790 | 4.15 ± 0.81 | t = -2.34,  p = 0.019 | 3.32 ± 0.92 | t = -8.19,  p < 0.001 | 3.50 ± 1.06 | t = -4.03,  p < 0.001 |
| Woman | 647 | 3.92 ± 0.81 |  | 3.04 ± 0.86 |  | 4.27 ± 0.76 |  | 3.79 ± 0.76 |  | 3.77 ± 0.95 |  |
| **Age category** | | | | | | | |  | | | |
| < 35 | 398 | 3.86 ± 0.85 | F = 1.79,  p = 0.129 | 3.00 ± 0.89 | Welch's F=2.19, p=.071 | 4.32 ± 0.75 | F = 5.55,  p <0.001 | 3.86 ± 0.74 | Welch's F=24.74, p<.001 | 3.85 ± 0.91 | Welch's F=11.00, p<.001 |
| 35 - 44 | 216 | 3.70 ± 0.87 |  | 3.14 ± 0.72 |  | 4.22 ± 0.77 |  | 2.96 ± 0.79 |  | 3.00 ± 1.19 |  |
| 45 - 54 | 183 | 3.90 ± 0.82 |  | 3.00 ± 0.87 |  | 4.23 ± 0.79 |  | 3.70 ± 0.83 |  | 3.71 ± 0.98 |  |
| 55 - 64 | 130 | 3.94 ± 0.77 |  | 3.03 ± 0.79 |  | 4.09 ± 0.75 |  | 3.58 ± 0.79 |  | 3.60 ± 1.02 |  |
| ≥65 | 45 | 4.02 ± 0.79 |  | 3.21 ± 0.77 |  | 3.83 ± 0.88 |  | 3.19 ± 0.94 |  | 3.33 ± 1.04 |  |
| **Relationship status** | | | | | | | |  | | | |
| Single | 175 | 3.92 ± 0.88 | t = 0.36,  p = 0.719 | 3.02 ± 0.86 | t = -0.37,  p = 0.715 | 4.19 ± 0.89 | t = -0.63,  p = 0.527 | 3.59 ± 0.87 | t = -0.66 ,  p = 0.509 | 3.65 ±1.06 | t = -0.41,  p = 0.680 |
| Relation | 825 | 3.90 ± 0.81 |  | 3.04 ± 0.84 |  | 4.23 ± 0.75 |  | 3.64 ± 0.84 |  | 3.68 ± 0.98 |  |
| **Parental status** | | | | | | | |  | | | |
| Yes | 582 | 3.90 ± 0.79 | t = -0.02,  p = 0.986 | 3.04 ± 0.80 | t = 0.22,  p = 0.829 | 4.16 ± 0.78 | t = -3.27,  p = 0.001 | 3.51 ± 0.88 | t = -5.68,  p < 0.001 | 3.55 ± 1.04 | t = -4.87,  p <0.001 |
| No | 415 | 3.90 ± 0.86 |  | 3.03 ± 0.89 |  | 4.32 ± 0.76 |  | 3.81 ± 0.77 |  | 3.85 ± 0.91 |  |
| **Physician type** | | | | | | | |  | | | |
| General practitioner^c^ | 339 | 3.61 ± 0.87 | t = -8.11,  p <0.001 | 2.96 ± 0.83 | t = -2.20,  p =0.028 | 4.00 ± 0.90 | t = -6.24,  p < 0.001 | 3.54 ± 0.87 | t = -2.33,  p = 0.020 | 3.70 ± 1.04 | t = 0.57,  p = 0.569 |
| Specialist | 645 | 4.06 ± 0.74 |  | 3.08 ± 0.85 |  | 4.35 ± 0.68 |  | 3.68 ± 0.83 |  | 3.66 ± 0.97 |  |
| **Career status** | | | | | | | |  | | | |
| Attending | 736 | 3.88 ± 0.82 | t = -2.13,  p = 0.034 | 3.08 ± 0.82 | t = 2.73,  p = 0.007 | 4.18 ± 0.79 | t = -3.67,  p < 0.001 | 3.55 ± 0.87 | t = -5.82,  p < 0.001 | 3.61 ± 1.03 | t = -4.30,  p < 0.001 |
| Resident | 249 | 4.00 ± 0.80 |  | 2.91 ± 0.90 |  | 4.37 ± 0.71 |  | 3.87 ± 0.72 |  | 3.89 ± 0.86 |  |
| **Weekly working hours category** | | | | | | | |  | | | |
| < 30 | 34 | 3.32 ± 0.92 | Welch's F = 83.94 ,  p <0.001 | 3.00 ± 0.87 | F = 2.26,  p = 0.080 | 3.79 ± 0.92 | Welch's F = 23.66 ,  p < 0.001 | 3.52 ± 0.83 | F = 3.60,  p = 0.013 | 3.32 ± 1.10 | F = 1.16,  p = 0.323 |
| 30 - 39 | 121 | 3.31 ± 0.76 |  | 2.92 ± 0.75 |  | 3.96 ± 0.81 |  | 3.42 ± 0.82 |  | 3.62 ± 1.01 |  |
| 40 - 50 | 452 | 3.76 ± 0.83 |  | 3.00 ± 0.83 |  | 4.14 ± 0.84 |  | 3.63 ± 0.85 |  | 3.71 ± 1.03 |  |
| >50 | 394 | 4.30 ± 0.60 |  | 3.12 ± 0.87 |  | 4.45 ± 0.60 |  | 3.70 ± 0.85 |  | 3.68 ± 0.95 |  |

**Note.** Values are means (SD). Two-sided p-values are reported. Independent-samples t-tests were used for two-group comparisons. Standard one-way ANOVAs were used for comparisons involving more than two groups when the homogeneity-of-variance assumption was met; Welch’s ANOVA was used when this assumption was violated. No post-hoc pairwise comparisons were conducted for these exploratory sub-dimension analyses. Following removal of item ec_10 from the revised 20-item HDMC model, Stigma towards Burnout was represented as a single three-item dimension and therefore had no sub-dimensions included in these analyses.

**Online supplemental table 4a**. HDMC dimension scores from the revised 20-item model according to working context among specialists (N=633).

|  | **N** | **Physicians’ Professional Commitment** | | **F (p)** | **Myth of the Invulnerable Physician** | | **F (p)** | **Stigma towards Burnout** | | **F (p)** |
| --- | --- | --- | --- | --- | --- | --- | --- | --- | --- | --- |
|  |  | **Mean** | **SD** |  | **Mean** | **SD** |  | **Mean** | **SD** |  |
| **Working context** |  |  |  | F = 0.69, p = 0.501 |  |  | F = 1.23, p = 0.293 |  |  | F = 2.05, p = 0.130 |
| Hospital setting | 560 | 3.89 | 0.59 |  | 3.67 | 0.75 |  | 2.60 | 0.85 |  |
| Private practice | 13 | 3.66 | 0.55 |  | 3.92 | 0.59 |  | 2.74 | 0.72 |  |
| Both | 60 | 3.72 | 0.54 |  | 3.58 | 0.69 |  | 2.38 | 0.75 |  |

**Note.** Values are means (SD). Of the 645 specialists included in the study, 633 had available data on working context. Two-sided p values are reported. Homogeneity of variance was assessed using Levene’s test; standard one-way ANOVAs were used for all comparisons.

**Online supplemental table 4b**. HDMC dimension scores from the revised 20-item model according to hospital type among specialists working in a hospital setting (N=619).

|  | **N** | **Physicians’ Professional Commitment** | | **F (p)** | **Myth of the Invulnerable Physician** | | **F (p)** | **Stigma towards Burnout** | | **F (p)** |
| --- | --- | --- | --- | --- | --- | --- | --- | --- | --- | --- |
|  |  | **Mean** | **SD** |  | **Mean** | **SD** |  | **Mean** | **SD** |  |
| **Hospital type** |  |  |  | F = 2.06, p = 0.128 |  |  | F = 0.60, p = 0.550 |  |  | F = 1.21, p = 0.299 |
| Public hospital | 204 | 3.73 | 0.60 |  | 3.71 | 0.70 |  | 2.50 | 0.84 |  |
| Private hospital | 127 | 3.77 | 0.60 |  | 3.64 | 0.77 |  | 2.62 | 0.83 |  |
| University hospital | 288 | 3.83 | 0.58 |  | 3.64 | 0.76 |  | 2.61 | 0.85 |  |

**Note**. Values are means (SD). Of the 620 specialists working in a hospital setting or in both hospital and private practice, hospital type was available for 619 participants (1 missing). Two-sided p values are reported. Homogeneity of variance was assessed using Levene’s test; standard one-way ANOVAs were used for all comparisons.

**Online supplemental table 5a**. HDMC dimension scores from the revised 20-item model according to work setting among general practitioners (N=339).

|  | **N** | **Physicians’ Professional Commitment** | | **t (p)** | **Myth of the Invulnerable Physician** | | **t (p)** | **Stigma towards Burnout** | | **t (p)** |
| --- | --- | --- | --- | --- | --- | --- | --- | --- | --- | --- |
|  |  | **Mean** | **SD** |  | **Mean** | **SD** |  | **Mean** | **SD** |  |
| **General medicine (consultation and home visits)** | | | | | | | | | | |
| No | 12 | 3.53 | 0.62 | t = 0.26,  p = 0.793 | 3.60 | 0.64 | t = 0.00,  p = 0.999 | 2.47 | 0.98 | t = 0.39,  p = 0.696 |
| Yes | 327 | 3.48 | 0.68 |  | 3.60 | 0.78 |  | 2.37 | 0.84 |  |
| **Hospital setting** | | | | | | | | | | |
| No | 325 | 3.47 | 0.68 | t = -0.88,  p = 0.380 | 3.59 | 0.78 | t = -0.87,  p = 0.384 | 2.37 | 0.84 | t = -1.31,  p = 0.192 |
| Yes | 14 | 3.64 | 0.63 |  | 3.77 | 0.58 |  | 2.67 | 0.91 |  |
| **Other institutions (e.g. family planning center, nursing home, ...)** | | | | | | | | | | |
| No | 287 | 3.47 | 0.67 | t = -0.82,  p = 0.413 | 3.58 | 0.80 | t = -0.67,  p = 0.502 | 2.35 | 0.84 | t = -1.37,  p = 0.171 |
| Yes | 52 | 3.55 | 0.74 |  | 3.66 | 0.66 |  | 2.53 | 0.88 |  |
| **Non-conventional medicine** | | | | | | | | | | |
| No | 328 | 3.48 | 0.68 | t = 0.08,  p = 0.934 | 3.62 | 0.76 | t = 2.82,  p = 0.005 | 2.38 | 0.82 | t = 0.03,  p = 0.973 |
| Yes | 11 | 3.46 | 0.79 |  | 2.95 | 0.85 |  | 2.36 | 1.39 |  |

**Note.** Values are means (SD). Two-sided p values are reported. Independent-samples t-tests were used for all comparisons. Homogeneity of variance was assessed using Levene’s test; unequal-variance estimates were used when this assumption was violated. Multiple responses regarding work setting were possible.

**Online supplemental table 5b**. HDMC dimension scores from the revised 20-item model according to type of practice among general practitioners (N=339).

|  | **N** | **Physicians’ Professional Commitment** | | **t (p)** | **Myth of the Invulnerable Physician** | | **t (p)** | **Stigma towards Burnout** | | **t (p)** |
| --- | --- | --- | --- | --- | --- | --- | --- | --- | --- | --- |
|  |  | **Mean** | **SD** |  | **Mean** | **SD** |  | **Mean** | **SD** |  |
| **Works alone** | | | | | | | | | | |
| No | 249 | 3.44 | 0.68 | t = -1.97,  p = 0.050 | 3.70 | 0.74 | t = 4.09,  p < 0.001 | 2.34 | 0.82 | t = -1.36,  p = 0.174 |
| Yes | 90 | 3.60 | 0 .66 |  | 3.31 | 0.81 |  | 2.48 | 0.81 |  |
| **Works in a monodisciplinary group** | | | | | | | | | | |
| No | 232 | 3.45 | 0.70 | t = -1.20,  p = 0.233 | 3.51 | 0.79 | t = -3.05,  p = 0.002 | 2.39 | 0.87 | t = 0.47,  p = 0.639 |
| Yes | 107 | 3.54 | 0.63 |  | 3.78 | 0.72 |  | 2.35 | 0.80 |  |
| **Works in a multidisciplinary group** | | | | | | | | | | |
| No | 189 | 3.56 | 0.65 | t = 2.40,  p = 0.017 | 3.56 | 0.80 | t = -0.94,  p = 0.350 | 2.40 | 0.86 | t = 0.60,  p = 0.550 |
| Yes | 150 | 3.38 | 0.71 |  | 3.64 | 0.74 |  | 2.35 | 0.83 |  |

**Note.** Values are means (SD). Two-sided p-values are reported. Homogeneity of variance was assessed using Levene’s test; the equal-variance assumption was met for all comparisons in this table, and standard independent-samples *t*-tests are therefore reported.

**Online supplemental table 6**. HDMC dimension scores from the revised 20-item model according to medical specialty among specialists (N=627).

| **Specialty** | **N** | **Physicians’ Professional Commitment** | | **The Myth of the Invulnerable Physician** | | **Stigma towards Burnout** | |
| --- | --- | --- | --- | --- | --- | --- | --- |
|  |  | **Mean** | **SD** | **Mean** | **SD** | **Mean** | **SD** |
| Internal medicine | 170 | 3.84 | 0.56 | 3.70 | 0.74 | 2.55 | 0.82 |
| Surgery | 66 | 3.89 | 0.57 | 3.58 | 0.79 | 2.87 | 0.85 |
| Acute medicine | 70 | 3.71 | 0.53 | 3.50 | 0.81 | 2.62 | 0.90 |
| Clinical pathology | 18 | 3.82 | 0.97 | 3.66 | 0.81 | 2.35 | 0.96 |
| Anaesthesiology | 60 | 3.74 | 0.53 | 3.53 | 0.62 | 2.66 | 0.72 |
| Dermatology | 13 | 3.37 | 0.34 | 3.58 | 0.47 | 2.38 | 0.59 |
| Obstetrics and gynaecology | 25 | 3.67 | 0.65 | 3.85 | 0.60 | 2.44 | 0.86 |
| Nuclear medicine | 8 | 3.68 | 0.76 | 3.77 | 0.69 | 2.46 | 1.02 |
| Physical medicine | 15 | 3.90 | 0.62 | 3.67 | 0.74 | 2.62 | 0.87 |
| Pediatry and neonatology | 54 | 3.73 | 0.55 | 3.78 | 0.53 | 2.44 | 0.89 |
| Ophtalmology | 10 | 4.06 | 0.66 | 3.62 | 0.58 | 2.07 | 0.56 |
| Otolaryngology | 18 | 3.65 | 0.53 | 3.84 | 0.79 | 2.43 | 0.61 |
| Psychiatry | 36 | 3.70 | 0.68 | 3.81 | 0.92 | 2.60 | 0.89 |
| Radiology | 12 | 3.78 | 0.39 | 3.40 | 1.02 | 2.81 | 0.69 |
| Palliative care | 2 | 3.59 | 0.96 | 4.50 | 0.47 | 2.67 | 0.94 |
| Neurology | 40 | 3.82 | 0.61 | 3.75 | 0.74 | 2.84 | 0.87 |
| Radiotherapy - oncology | 5 | 4.11 | 0.45 | 3.73 | 0.87 | 2.00 | 1.22 |
| Other | 5 | 4.00 | 1.18 | 3.83 | 1.00 | 2.00 | 1.15 |

**Note.** Values are means (SD). Medical specialty was available for 627 specialists. Given the highly unequal representation and small sample sizes across specialty groups, no inferential comparisons were conducted; descriptive statistics are presented for exploratory purposes only. Scores are based on the revised 20-item HDMC model.

**Online supplemental table 7**. Sensitivity analyses comparing continuous, binary, and detailed categorical specifications of age and weekly working hours in multivariable regression models of HDMC dimension scores from the revised 20-item model (N=940 for continuous and binary specifications; N=939 for detailed categorical specifications).

| **Outcome** | **Specification** | **Variable / contrast** | **B (95% CI)** | **p-value** | **Adjusted R²** |
| --- | --- | --- | --- | --- | --- |
| **Physicians’ Professional Commitment** | **Continuous** | Age (per year) | 0.003 (−0.002 to 0.007) | 0.248 | 0.123 |
|  |  | Weekly working hours (per hour/week) | 0.015 (0.012 to 0.019) | <0.001 |  |
|  | **Binary** | Age ≥45 years (ref: <45 years) | 0.038 (−0.060 to 0.136) | 0.448 | 0.108 |
|  |  | >50 h/week (ref: ≤50 h/week) | 0.337 (0.251 to 0.422) | <0.001 |  |
|  | **Detailed categorical** | Age 35–44 years (ref: <35 years) | 0.004 (−0.134 to 0.141) | 0.959 | 0.117 |
|  |  | Age 45–54 years (ref: <35 years) | 0.013 (−0.136 to 0.16) | 0.863 |  |
|  |  | Age 55–64 years (ref: <35 years) | 0.0537 (−0.102 to 0.216) | 0.485 |  |
|  |  | Age ≥65 years (ref: <35 years) | 0.099 (−0.127 to 0.324) | 0.390 |  |
|  |  | 30–39 h/week (ref: <30 h/week) | −0.033 (−0.284 to 0.218) | 0.797 |  |
|  |  | 40–50 h/week (ref: <30 h/week) | 0.206 (−0.027 to 0.440) | 0.083 |  |
|  |  | >50 h/week (ref: <30 h/week) | 0.494 (0.256 to 0.732) | <0.001 |  |
| **Myth of the Invulnerable Physician** | **Continuous** | Age (per year) | −0.019 (−0.024 to −0.014) | <0.001 | 0.150 |
|  |  | Weekly working hours (per hour/week) | 0.005 (0.001 to 0.009) | 0.012 |  |
|  | **Binary** | Age ≥45 years (ref: <45 years) | −0.295 (−0.410 to −0.180) | <0.001 | 0.120 |
|  |  | >50 h/week (ref: ≤50 h/week) | 0.083 (−0.017 to 0.183) | 0.104 |  |
|  | **Detailed categorical** | Age 35–44 years (ref: <35 years) | −0.126 (−0.285 to 0.033) | 0.121 | 0.148 |
|  |  | Age 45–54 years (ref: <35 years) | −0.204 (−0.377 to −0.032) | 0.020 |  |
|  |  | Age 55–64 years (ref: <35 years) | −0.556 (−0.740 to −0.371) | <0.001 |  |
|  |  | Age ≥65 years (ref: <35 years) | −0.680 (−0.941 to −0.419) | <0.001 |  |
|  |  | 30–39 h/week (ref: <30 h/week) | −0.210 (−0.500 to 0.080) | 0.156 |  |
|  |  | 40–50 h/week (ref: <30 h/week) | −0.049 (−0.320 to 0.222) | 0.723 |  |
|  |  | >50 h/week (ref: <30 h/week) | 0.022 (−0.253 to 0.298) | 0.873 |  |
| **Stigma towards Burnout** | **Continuous** | Age (per year) | −0.009 (−0.015 to −0.003) | 0.003 | 0.054 |
|  |  | Weekly working hours (per hour/week) | 0.007 (0.002 to 0.011) | 0.002 |  |
|  | **Binary** | Age ≥45 years (ref: <45 years) | −0.222 (−0.356 to −0.089) | <0.001 | 0.051 |
|  |  | >50 h/week (ref: ≤50 h/week) | 0.154 (0.038 to 0.270) | 0.009 |  |
|  | **Detailed categorical** | Age 35–44 years (ref: <35 years) | −0.217 (−0.405 to −0.029) | 0.024 | 0.053 |
|  |  | Age 45–54 years (ref: <35 years) | −0.343 (−0.546 to −0.140) | 0.001 |  |
|  |  | Age 55–64 years (ref: <35 years) | −0.440 (−0.658 to −0.223) | <0.001 |  |
|  |  | Age ≥65 years (ref: <35 years) | −0.292 (−0.600 to 0.015) | 0.063 |  |
|  |  | 30–39 h/week (ref: <30 h/week) | 0.091 (−0.434 to 0.251) | 0.600 |  |
|  |  | 40–50 h/week (ref: <30 h/week) | 0.052 (−0.371 to 0.268) | 0.751 |  |
|  |  | >50 h/week (ref: <30 h/week) | 0.107 (−0.218 to 0.431) | 0.519 |  |

**Note.** B, unstandardised regression coefficient; CI, confidence interval; HDMC, Harmful Dimensions of Medical Culture. All models were adjusted for gender, relationship status, parental status, physician type (general practitioner vs specialist), and physician status (attending physician vs resident). The continuous specification corresponds to the primary analyses reported in Table 4. In sensitivity analyses, age and weekly working hours were alternatively entered as binary or detailed categorical variables. In the binary specification, the reference categories were <45 years for age and ≤50 hours/week for weekly working hours. In the detailed categorical specification, the reference categories were <35 years for age and <30 hours/week for weekly working hours. Continuous coefficients represent the estimated difference in the outcome associated with a one-year increase in age or a one-hour/week increase in weekly working hours. Missing values were excluded listwise. All p-values are two-sided. Sample sizes were 940 for the continuous and binary specifications and 939 for the detailed categorical specification.

**Online supplemental figure 3. Measurement model of the revised 20-item Harmful Dimensions of Medical Culture (HDMC)**

**Note.** The revised 20-item HDMC measurement model comprises three correlated dimensions. Physicians’ Professional Commitment and the Myth of the Invulnerable Physician are represented as second-order latent dimensions measured through three and two first-order factors, respectively. Following removal of item ec_10, Stigma towards Burnout is represented directly as a latent factor measured by the three remaining items (ec_5, ec_6, and ec_9), without the previously specified Colleagues’ Stigma and Personal Stigma first-order factors.

**Online supplemental table 8**. Measurement invariance testing of the revised 20-item HDMC model across physician subgroups

| **Comparison** | **Model** | **χ² (df)** | **CFI** | **RMSEA** | **SRMR** | **AIC** | **BIC** | **ΔCFI** | **ΔRMSEA** | **ΔSRMR** |
| --- | --- | --- | --- | --- | --- | --- | --- | --- | --- | --- |
| **Gender** | **Configural** | 934.215 (324) | .920 | .062 | .056 | 50718.227 | 51384.863 | — | — | — |
|  | **Metric** | 960.468 (341) | .919 | .060 | .061 | 50710.480 | 51293.787 | −.001 | −.002 | +.005 |
|  | **Scalar** | 1080.079 (361) | .906 | .063 | .063 | 50790.091 | 51275.363 | −.013 | +.003 | +.002 |
|  | **Partial scalar¹** | 1035.477 (360) | .912 | .061 | .062 | 50747.489 | 51237.662 | −.007 | +.001 | +.001 |
| **Age (<45 vs ≥45 years)** | **Configural** | 829.306 (324) | .933 | .057 | .058 | 49283.862 | 49947.454 | — | — | — |
|  | **Metric** | 848.803 (341) | .932 | .055 | .060 | 49269.358 | 49850.002 | −.001 | −.002 | +.002 |
|  | **Scalar** | 1106.542 (361) | .901 | .065 | .064 | 49487.097 | 49970.154 | −.031 | +.010 | +.004 |
|  | **Partial scalar²** | 942.573 (356) | .922 | .058 | .061 | 49333.129 | 49840.582 | −.010 | +.003 | +.001 |
| **Resident vs attending** | **Configural** | 864.013 (324) | .929 | .058 | .059 | 50161.456 | 50826.855 | — | — | — |
|  | **Metric** | 879.528 (341) | .929 | .057 | .061 | 50142.863 | 50725.087 | +.001 | −.001 | +.002 |
|  | **Scalar** | 1094.826 (361) | .903 | .064 | .067 | 50318.710 | 50803.081 | −.026 | +.007 | +.006 |
|  | **Partial scalar³** | 958.382 (358) | .921 | .058 | .063 | 50187.803 | 50686.852 | −.008 | +.001 | +.002 |
| **GP vs specialist** | **Configural** | 912.354 (324) | .921 | .061 | .057 | 50162.414 | 50827.813 | — | — | — |
|  | **Metric** | 939.302 (341) | .920 | .060 | .062 | 50155.362 | 50737.586 | −.001 | −.001 | +.005 |
|  | **Scalar** | 1093.309 (361) | .902 | .064 | .070 | 50269.368 | 50753.740 | −.018 | +.004 | +.008 |
|  | **Partial scalar⁴** | 1031.398 (358) | .910 | .062 | .070 | 50213.457 | 50712.506 | −.010 | +.002 | +.008 |

**Note** : HDMC, Harmful Dimensions of Medical Culture; GP, general practitioner; CFI, comparative fit index; RMSEA, root mean square error of approximation; SRMR, standardized root mean square residual; AIC, Akaike information criterion; BIC, Bayesian information criterion. The revised 20-item model excluded item ec_10 from the Stigma towards Burnout dimension, which was therefore represented directly by its three remaining items (ec_5, ec_6, and ec_9). Metric invariance was evaluated relative to the configural model. Full and partial scalar invariance were evaluated relative to the metric model. Decreases in CFI ≤0.010 and increases in RMSEA ≤0.015 were considered supportive of invariance; increases in SRMR ≤0.030 were used for metric invariance and ≤0.010 for scalar invariance. Partial scalar invariance was supported after freeing the intercepts of: ¹ rc_6 for gender; ² rc_6, vc_10, rc_5, vc_3, and ec_5 for age; ³ rc_6, vc_10, and rc_5 for resident versus attending physicians; and ⁴ rc_5, rc_3, and ec_14 for GP versus specialist physicians. Δ indices reported for the full scalar and final partial scalar models are relative to the metric model.

**Online supplemental appendix 1.**

We followed Field’s guidelines for assessing the assumptions and diagnostics of multiple linear regression models (1). For each regression model, we conducted the following checks:

- **Independence of errors** was assessed using the Durbin-Watson statistic. Values close to 2 and within the prespecified acceptable range of 1-3 were considered satisfactory. Durbin-Watson statistics ranged from 1.963 to 2.111 across the three models.
- **Multicollinearity** was evaluated through Variance Inflation Factors (VIFs). All VIFs were below 4 and ranged from 1.091 to 2.120, indicating no concerning multicollinearity.
- **Casewise diagnostics** included examination of standardized residuals, Cook’s distance, leverage, and Mahalanobis distance. Standardized residuals were examined against thresholds of |2|, |2.5|, and |3|. Across models, fewer than 5% of cases had absolute standardized residuals greater than 2. A small number of cases exceeded |2.5| or |3|; these were retained because there was no evidence that they exerted undue influence on the regression estimates. Cook’s distances were examined using a threshold of 1, leverage values relative to the average leverage, and Mahalanobis distances using a threshold of 25. No case had a Cook’s distance greater than 1. Two cases exceeded a Mahalanobis distance of 25, but neither exceeded 30 and neither showed evidence of undue influence.
- **Linearity and homoscedasticity** were assessed by visual inspection of plots of residuals against predicted values. No substantial systematic pattern or pronounced funnel shape indicating problematic non-linearity or heteroscedasticity was observed.
- **Normality of residuals** was assessed by visual inspection of histograms and normal Q-Q plots of the regression residuals, which did not indicate substantial departures from normality.

1.	Field A. Discovering statistics using SPSS: and sex, drugs and rock “n” roll. 3rd ed. Los Angeles: SAGE Publications; 2009. 821 p.
